# Factors associated with child stunting, underweight, and wasting in eight Latin American and Caribbean countries and regions: a regional analysis using Multiple Indicator Cluster Surveys

**DOI:** 10.7189/jogh.16.04201

**Published:** 2026-07-17

**Authors:** Xiaoping Zhang, Yawen Deng, Tingting Xie, Sizhe Gao, Zhongbin Tao, Fei Teng, Guiying Hu, Feng Wang, Xiaohui Wang, Guanlan Zhao

**Affiliations:** 1 School of Public Health, Lanzhou University, Lanzhou, China; 2 Department of Paediatrics, The First Hospital of Lanzhou University, Lanzhou, China; 3 Department of Obstetrics and Gynaecology, The First Hospital of Lanzhou University, Lanzhou, China; 4 School of Public Administration, Hangzhou Normal University, Hangzhou, China; 5 Public Health Research Group, University of Alicante, Alicante, Spain

**Keywords:** stunting, wasting, underweight, child undernutrition, Latin America and the Caribbean

## Abstract

**Background:**

Although child undernutrition in Latin America and the Caribbean is lower than the global average, the region still faces substantial challenges in achieving Sustainable Development Goal 2.2. We aimed to identify factors associated with child undernutrition to inform efforts in this region to improve child nutrition and reduce health inequities.

**Methods:**

We analysed 34,862 children aged 0–59 months using Multiple Indicator Cluster Surveys from eight Latin American and Caribbean countries. We fitted multivariable logistic regression models accounting for complex survey design to assess associations between selected factors and three indicators of undernutrition.

**Results:**

Prevalence of stunting, underweight, and wasting was 11.0, 4.4, and 2.8%, respectively. Compared with children aged 0–11 months, stunting was more common at 12–23 months (odds ratio (OR) = 1.55; 95% confidence interval (CI) = 1.30, 1.84). Stunting was less common among children whose mothers had higher education (OR = 0.62; 95% CI = 0.51, 0.75) and those from the richest households (OR = 0.59; 95% CI = 0.47, 0.76). Underweight was less common among children ever breastfed (OR = 0.50; 95% CI = 0.34, 0.74, in 0–23 months subgroup) and those whose mothers had higher education (OR = 0.48; 95% CI = 0.35, 0.67). Compared with the youngest group, wasting was less common in older children, *e.g*. 12–23 months (OR = 0.64; 95% CI = 0.47, 0.86). In subgroup analyses, maternal health insurance was associated with lower odds of wasting in seven-country analyses excluding Cuba (OR = 0.74; 95% CI = 0.56, 0.97).

**Conclusions:**

Child undernutrition in Latin America and the Caribbean reflects persistent socioeconomic inequities. These findings support policies that invest in girls’ education, target nutrition interventions to the first 1000 days of life, integrate nutrition services into maternal and child health programmes, and address the emerging challenges of the nutrition transition.

Undernutrition, defined as a deficiency in energy or nutrient intake or absorption, leads to impaired physical growth, low body weight, and developmental delays, and in children under five, it primarily manifests as stunting, underweight, and wasting, which reflect chronic growth restriction, low weight for age, and acute weight loss, respectively [1]. Undernutrition has profound and lasting effects on children’s health and social development [2,3]. At the individual level, undernutrition in the short term impairs immune function, weakens disease resistance, and increases the risk of infection and mortality [4]. In the long term, undernutrition during early childhood often results in irreversible health and developmental consequences [5]. Chronic undernutrition in infancy leads to stunted growth and impairs brain development, resulting in reduced cognitive function, poor academic performance, and limited future income and social mobility [6]. Furthermore, early childhood undernutrition is associated with an increased risk of chronic diseases in adulthood, including metabolic conditions such as obesity, diabetes, and hypertension [7]. At the societal level, child undernutrition undermines economic development and social progress by reducing the population’ s overall human capital [3]. Widespread child undernutrition reduces national economic productivity while disproportionately affecting disadvantaged populations, thereby exacerbating health and economic inequalities in a self-perpetuating cycle [5]. Therefore, improving children’s nutritional status is essential not only for individual health but also for advancing social equity and sustainable economic development [8].

In 2022, an estimated 22.3% (148 million) of children under five globally were stunted, 6.8% (45 million) were wasted, and 2.1% (13.6 million) were severely wasted, and despite some progress since 2012, undernutrition remains a major global public health challenge for children [9]. In Latin America and the Caribbean, undernutrition among children under five remains a serious concern. In 2022, the regional wasting rate was 1.4%, well below the global average; however, certain countries still experienced relatively high rates, such as 6.5% in Guyana and 3.7% in Haiti. Regarding stunting, approximately 5.7 million children in the region were affected, accounting for 11.5%. In some countries, prevalence remains significantly higher than the global average; for example, the stunting rate in Honduras is as high as 23%. Although childhood stunting has declined since 2000, the pace of reduction has slowed in recent years: from 2012 to 2022, prevalence fell by only 1.2 percentage points, and remained nearly stable from 2020 to 2022 (from 11.7 to 11.5%) [10]. Given the stagnation observed in recent years, achieving the Sustainable Development Goal 2.2 of eliminating all forms of undernutrition by 2030 will necessitate intensified and sustained efforts across the region [11].

Children’s nutritional status is influenced by multiple biological and social factors, including individual characteristics, maternal health and health care utilisation, and family socioeconomic conditions [5]. At the individual level, biological traits such as age and sex may influence children’s nutritional needs and susceptibility to undernutrition [12,13]. Infectious diseases, such as diarrhoea, fever, and respiratory infections can impair children’s short-term nutritional status by increasing energy demands and reducing nutrient absorption efficiency [14]. Breastfeeding history and mode of delivery may also have long-term impacts on development through their effects on early nutritional intake [12]. Additionally, maternal factors such as health status, education, age, access to health insurance, and use of perinatal services (such as prenatal care and breastfeeding counselling) can influence child nutrition by supporting foetal growth and caregiving practices [14,15]. Family socioeconomic status serves as a foundational determinant, with household wealth directly linked to health care access, and potentially affecting children’s risk of infection and quality of care through living and sanitation conditions [16,17].

A systematic analysis of these factors can provide a scientific foundation for targeted interventions. Periodic re-evaluation of regional evidence is essential to ensure that policies remain responsive to shifting socio-economic contexts; indeed, previous study have consistently emphasised the need for continuous monitoring to address persistent data gaps [18]. While other data sources may exist, the sixth round of the Multiple Indicator Cluster Surveys (MICS) currently represents one of the most recent, harmonised, and standardised data sets available for a multi-country analysis of child undernutrition in the Latin America and the Caribbean region. By utilising the sixth round of the MICS (MICS6) to identify these correlates, this study aims to provide a scientific basis for tailoring localised health policies, advancing child health equity, and contributing to the monitoring of Sustainable Development Goal 2.2.

## METHODS

This study followed the Reporting of Observational Studies in Epidemiology (STROBE) guidelines for cross-sectional studies [19]. The completed STROBE checklist is provided in Checklist S1 in the **Online Supplementary Document**.

### Study design and data source

This study was a cross-sectional secondary analysis of nationally representative household survey data from MICS6, conducted by UNICEF between 2017–2020 across eight countries and regions in Latin America and the Caribbean, namely Argentina, the Dominican Republic, Costa Rica, Cuba, Guyana, Honduras, Suriname, and the Turks and Caicos Islands.

The MICS is a globally standardised household survey programme designed to monitor health, nutrition, and socioeconomic indicators for women and children [20]. A two-stage stratified cluster sampling design was employed in MICS6 to ensure national and subnational representativeness. Enumeration areas were first selected with probability proportional to size, after stratification by region and urban-rural status, followed by the random selection of 20–25 households per enumeration area. Sample weights were provided to account for non-response and the complex survey design [20]. Furthermore, to enhance statistical power for low-prevalence indicators, the MICS has included additional sampling of households with children under five years old since the third round. During the listing phase, households with children are identified and given a higher probability of selection in the second stage, improving the representativeness of child-related data [21].

### Study population and sampling size

This study focussed on Latin American and Caribbean countries and regions included in MICS6 with complete anthropometric data. Countries or regions were excluded if they had missing anthropometric measurements (*e.g*. Jamaica and Trinidad and Tobago) or if their data sets were not publicly available (*e.g*. Nicaragua).

To construct the analytical sample, we merged the women’s data set (n = 92,124) and the under-five children’s data set (n = 40,481) from MICS6 using household and individual identifiers, yielding 40,481 matched mother–child pairs. Children aged 0–59 months were included if they met three criteria:

(1) completed questionnaires

(2) non-missing and plausible Z-scores for all three anthropometric indicators: height-for-age, weight-for-age, and weight-for-height

(3) mothers not classified under Argentina’s ‘special education’ category (n = 10 excluded for cross-national comparability).

**Figure 1:**
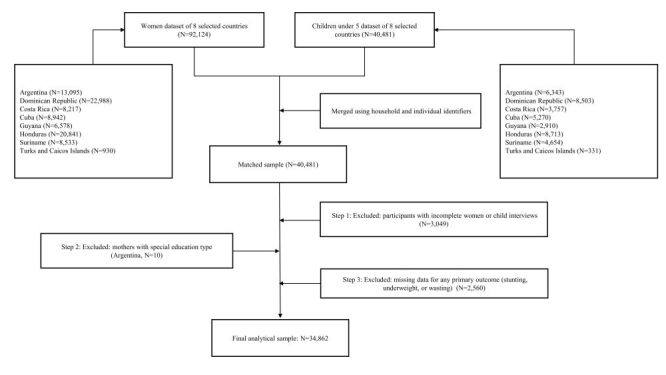
Flow diagram illustrating sample selection and exclusions, based on MICS6 data from eight countries or regions in Latin America and the Caribbean.

After sequential exclusions, incomplete interviews (n = 3,049), special education cases (n = 10), and missing outcome data (n = 2,560), the final analytical sample comprised 34,862 children: Argentina (n = 6,343), Dominican Republic (n = 8,503), Costa Rica (n = 3,757), Cuba (n = 3,271), Guyana (n = 2,910), Honduras (n = 8,713), Suriname (n = 4,654), and Turks and Caicos Islands (n = 331) (**Figure 1**).

### Exposures

All variables were sourced from the standardised MICS questionnaires. Guided by the UNICEF conceptual framework on determinants of maternal and child nutrition and supported by previous studies [13–15,22], we systematically selected variables across three domains:

(1) enabling factors (maternal education, marital status, utilisation of prenatal care, receipt of breastfeeding guidance after delivery, and health insurance coverage)

(2) underlying factors (household wealth index and safe disposal of child’s stool [22])

(3) immediate factors (recent illness history of the child, including diarrhoea, fever, and cough, alongside breastfeeding history).

In addition, key demographic variables, such as child age, child sex, and maternal age, were incorporated to evaluate their associations with child nutritional outcomes and adjust for baseline biological differences.

To address potential structural missingness and selection bias, we explicitly partitioned these selected variables into core main-model factors and age/country-restricted factors (**Table 1**). Core variables, applicable to the entire 0–59 months cohort, included demographic characteristics, maternal education, marital status, mother’s alcohol consumption history, deceased children history, household wealth quintile, and immediate illness history. Variables with strict applicability constraints, such as ever breastfed, safe disposal of child’s stool, caesarean delivery, antenatal care, and postnatal breastfeeding guidance, were excluded from the main pooled models to prevent structural confounding. Instead, they were evaluated in specific age-restricted subgroup analyses. Similarly, health insurance coverage was assigned to Panel B of **Table 1** due to country-level structural missingness. Notably, because Cuba implements a universal free medical care system, the MICS survey did not collect health insurance data for this country. To avoid deterministic bias (zero variance) and arbitrary classification, Cuba was excluded from the specific subgroup analyses that evaluated health insurance variables.

**Table 1:** Definitions and categorisation of the variables included in the main and supplementary analyses*

Factor	Definition	Category	Reference group
**Panel A:** **core variables**			
Children’s age	Represents the age category of the child in months, grouped into five developmental bands	0–11, 12–23, 24–35, 36–47, 48–59	0–11
Children’s sex	Biological sex of the child	Boy, Girl	Boy
Diarrhoea in past two weeks	Whether the child had diarrhoea in the last two weeks	Yes, No	No
Fever in past two weeks	Whether the child had fever in the last two weeks	Yes, No	No
Cough in past two weeks	Whether the child had a cough in the last two weeks	Yes, No	No
Mother’s age	Age group of the mother at the time of interview	15–19, 20–34, 35–49	20–34
Mother’s education level	The highest level of formal education completed by the mother	Primary or none, secondary, higher	Primary or none
Mother’s marital or cohabitation status	Whether the mother is currently married or cohabiting	Yes, No	No
Mother ever consumed alcohol	Whether the mother has ever consumed alcohol	Yes, No	No
Whether the mother has deceased children	Denotes whether the mother has had a live-born child who later died	Yes, No	No
Household wealth quintile	Ranks household wealth into quintiles based on asset ownership and living conditions, serving as a proxy for socioeconomic status	Poorest, Poorer, Middle, Richer, Richest	Poorest
**Panel B:** **variables restricted to subgroup and sensitivity analyses**			
Ever breastfed	Whether the child has ever been breastfed	Yes, No	No
Safe disposal of child’s stool	Whether child’s faeces were hygienically disposed	Yes, No	No
caesarean delivery	Whether the child was delivered by caesarean section	Yes, No	No
Received antenatal care	Whether the mother received any antenatal checkups during her pregnancy with the child	Yes, No	No
Breastfeeding guidance after delivery	Whether a health professional provided the mother with breastfeeding counselling within 48 h after delivery	Yes, No	No
Child health insurance coverage	Whether the child has health insurance	Yes, No	No
Mother’s health insurance coverage	Whether the mother has health insurance	Yes, No	No

*Binary variables were coded as 1 for ‘Yes’ and 0 for ‘No’. Sex was coded as 1 for boys and 0 for girls. Multi-category variables were coded sequentially according to their categories as presented in the table. Reference groups for regression analyses are indicated in the rightmost column. Due to structural missingness, the variables in Panel B were excluded from the main models (Panel A) to avoid bias. Instead, they were evaluated in specific age-restricted subgroups or country-specific sensitivity analyses.

Furthermore, to ensure statistical transparency and properly model nonlinear epidemiological relationships, continuous variables were categorised. Modelling child age as a continuous variable assumes a constant linear effect, which contradicts the well-documented nonlinear trajectory of child growth. Therefore, child age was grouped into 12-month bands. This approach accurately captures distinct developmental vulnerabilities (such as the peak risk periods during the complementary feeding transition) and directly aligns with standard World Health Organization reporting age bands, thereby facilitating cross-study comparability and age-targeted policy design. Similarly, maternal age was categorised into 15–19 years, 20–34 years, and 35–49 years. These specific brackets correspond to adolescent mothers, mothers of optimal childbearing age, and high-risk mothers of advanced age, respectively. This categorisation effectively captures the unique obstetric and socioeconomic risks tied to early and late pregnancies, which would otherwise be obscured in a simple linear specification.

### Outcomes

The outcome variables in this study were three standard indicators of child undernutrition: stunting, underweight, and wasting, defined according to WHO growth reference standards [23]. These indicators were assessed among children aged 0–59 months. Stunting was identified by a height-for-age z-score<−2 standard deviations (SD), indicating chronic linear growth retardation. Underweight was defined as a weight-for-age z-score<−2 SD, reflecting low body weight relative to age. Wasting, indicative of acute malnutrition, was defined as a weight-for-height z-score<−2 SD. All z-scores were calculated by comparing individual anthropometric measurements to the median values of a healthy reference population of the same age and sex.

### Statistical analysis

The data cleaning and harmonisation were performed using SPSS version 29.0 (IBM Corp., Armonk, NY, USA). All statistical analyses were conducted in *R*, version 4.4.2 (*R* Foundation for Statistical Computing, Vienna, Austria). To ensure cross-national comparability, only variables consistently available across all national surveys were retained, and categories and measurement units were standardised.

We accounted for the complex sampling design of the MICS surveys using the survey package in *R*. Primary sampling units (PSU), stratification variables, and sampling weights were incorporated in all analyses [20]. Regarding the weights, we implemented within-country weight rescaling to prevent countries with larger sample sizes from disproportionately dominating the pooled results. For variance estimation, we strictly preserved and utilised the original PSU and strata information to ensure the correct calculation of standard errors. For Suriname, which lacked predefined stratification variables, we constructed a stratification variable based on its 10 administrative divisions and three geographic region types (urban, coastal rural, inland rural), with the cluster identifier serving as the primary sampling unit [24].

Descriptive statistics were performed on the unimputed data, with frequencies and percentages calculated for key variables. Weighted prevalence estimates and 95% confidence intervals (CI) for nutritional outcomes were estimated by accounting for these complex survey design features. Missing data in the multivariable models were handled according to their mechanisms (Table S1 in the **Online Supplementary Document**). For item non-response in core factors, we employed multiple imputation by chained equations (MICE), generating 20 imputed data sets with a maximum of 10 iterations and setting a random seed of 2,026 for reproducibility (m = 20, maxit = 10, seed = 2,026). To maintain the weighted and hierarchical data structure, the imputation model included the rescaled weights and the country indicator. The model incorporated 15 variables to support the Missing at Random assumption: three nutritional outcomes and 12 core predictors (child age, sex, recent diarrhoea, fever, cough, mother’s age, education, marital status, maternal alcohol consumption, history of child death, household wealth, and country). To prevent non-convergence due to high dimensionality, PSU and stratification were excluded from the predictor matrix. Imputation quality was confirmed via trace plots; for the ‘Whether the mother has deceased children’ variable, fluctuations were attributed to discrete assignment effects in a binary space given its extremely low missingness rate (<0.1%) (Figure S1 in the **Online Supplementary Document**). For analyses targeting variables with specific structural missingness (Table S1, Panels B and C in the **Online Supplementary Document**), the imputation models were extended to include these factors within their respective age-restricted or country-restricted analytical samples.

For each outcome (stunting, underweight, wasting), we fitted separate multivariable binary logistic regression models, reporting odds ratios (OR) and 95% CIs. Multicollinearity was assessed using the generalised variance inflation factor (GVIF). For all predictors, the adjusted GVIF values (GVIF^^(1/(2*Df))^) were <2, indicating the absence of significant collinearity (Table S2 in the **Online Supplementary Document**).

To evaluate result stability, we conducted the following:

(1) calculating prevalence ratios (PR) via modified Poisson regression to provide more conservative estimates for stunting given its prevalence exceeded 10%, while also estimating PRs for underweight and wasting to maintain methodological consistency across all outcomes

(2) constructing age-restricted subgroup models for children aged 0–35 months and 0–23 months to assess predictors with structural missingness, such as breastfeeding and clinical care indicators

(3) performing analyses excluding Cuba to assess health insurance effects and excluding the Turks and Caicos Islands to minimise potential bias from the small sample size

(4) conducting multiplicative interaction tests (reported as Ratio of OR)

(5) performing country-stratified regression analyses to visualise the consistency of associations across the region.

## RESULTS

This study included 34,862 children aged 0–59 months from eight countries in Latin America and the Caribbean. Overall, the prevalence of stunting, underweight, and wasting was 11.0% (95% CI = 10.7, 11.3), 4.4% (95% CI = 4.2, 4.7), and 2.8% (95% CI = 2.6, 2.9), respectively (**Table 2**). The prevalence of malnutrition varied across countries (Table S3 in the **Online Supplementary Document**). Honduras had the highest prevalence of stunting at 20.3% (95% CI = 19.4, 21.2). Underweight was highest in Guyana at 7.3%, and wasting was highest in Guyana and Suriname at 5.9%.

**Table 2:** Prevalence of stunting, underweight, and wasting by selected factors among children aged 0–59 mo in eight countries or regions of Latin America and the Caribbean*

Factor	Children observed n, %	Prevalence, % (95% CI)		
		**Stunting**	**Underweight**	**Wasting**
**Total**	34,862 (100.0)	11.0 (10.7, 11.3)	4.4 (4.2, 4.7)	2.8 (2.6, 2.9)
Children’s age				
*0–11 mo*	6,289 (18.0)	9.3 (8.5, 10.0)	4.4 (3.9, 4.9)	3.6 (3.2, 4.1)
*12–23 mo*	7,106 (20.4)	12.6 (11.8, 13.3)	4.3 (3.8, 4.8)	2.8 (2.4, 3.2)
*24–35 mo*	6,902 (19.8)	12.2 (11.5, 13.0)	4.7 (4.2, 5.2)	2.3 (2.0, 2.7)
*36–47 mo*	7,467 (21.4)	11.5 (10.7, 12.2)	4.5 (4.0, 5.0)	2.5 (2.2, 2.9)
*48–59 mo*	7,098 (20.4)	9.3 (8.7, 10.0)	4.3 (3.8, 4.7)	2.6 (2.2, 2.9)
Children’s sex				
*Female*	17,115 (49.1)	10.0 (9.6, 10.5)	3.9 (3.6, 4.2)	2.4 (2.2, 2.6)
*Male*	17,747 (50.9)	11.9 (11.5, 12.4)	4.9 (4.6, 5.2)	3.1 (2.8, 3.3)
Diarrhoea in past two weeks				
*Yes*	4,140 (11.9)	10.9 (9.9, 11.8)	4.8 (4.1, 5.4)	2.9 (2.4, 3.4)
*No*	30,805 (88.6)	11.0 (10.7, 11.4)	4.4 (4.2, 4.6)	2.7 (2.6, 2.9)
*Missing*	83 (0.2)	18.1 (9.8, 26.4)	7.2 (1.7, 12.8)	1.2 (0.0, 3.6)
Fever in past two weeks				
*Yes*	6,695 (19.2)	11.3 (10.5, 12.0)	5.7 (5.2, 6.3)	3.4 (3.0, 3.8)
*No*	28,230 (81.1)	10.9 (10.6, 11.3)	4.1 (3.9, 4.4)	2.6 (2.4, 2.8)
*Missing*	63 (0.2)	12.7 (4.5, 20.9)	4.8 (0.0, 10.0)	0.0 (0.0, 0.0)
Cough in past two weeks				
*Yes*	8,326 (23.9)	10.7 (10.1, 11.4)	5.1 (4.6, 5.5)	2.8 (2.4, 3.1)
*No*	26,589 (76.4)	11.1 (10.7, 11.5)	4.2 (4.0, 4.5)	2.7 (2.5, 2.9)
*Missing*	53 (0.2)	9.4 (1.6, 17.3)	1.9 (0.0, 5.5)	3.8 (0.0, 8.9)
Mother’s age				
*15–19 y*	2,279 (6.5)	13.5 (12.1, 14.9)	4.9 (4.0, 5.8)	3.6 (2.8, 4.3)
*20–34 y*	25,148 (72.1)	10.8 (10.4, 11.1)	4.3 (4.1, 4.6)	2.6 (2.4, 2.8)
*35–49 y*	7,435 (21.3)	11.1 (10.4, 11.8)	4.6 (4.1, 5.1)	2.9 (2.5, 3.3)
Mother’s education level				
*Primary or none*	9,604 (27.5)	20.5 (17.7, 23.3)	7.9 (6.0, 9.7)	3.1 (1.9, 4.3)
*Secondary*	17,218 (49.4)	8.9 (8.5, 9.3)	3.8 (3.5, 4.1)	3.2 (2.9, 3.4)
*Higher*	7,248 (20.8)	5.6 (5.1, 6.1)	2.1 (1.8, 2.4)	2.2 (1.9, 2.5)
*Missing*	802 (2.3)	0.0 (0.0, 0.0)	0.0 (0.0, 0.0)	0.0 (0.0, 0.0)
Experienced child death				
*Yes*	1,527 (4.4)	16.6 (14.7, 18.4)	6.7 (5.5, 8.0)	3.0 (2.2, 3.9)
*No*	33,323 (95.6)	10.7 (10.4, 11.1)	4.3 (4.1, 4.5)	2.7 (2.6, 2.9)
*Missing*	12 (0.0)	25.0 (0.5, 49.5)	0.0 (0.0, 0.0)	0.0 (0.0, 0.0)
Mother ever consumed alcohol				
*Yes*	19,325 (55.4)	8.1 (7.7, 8.5)	3.6 (3.3, 3.9)	2.7 (2.4, 2.9)
*No*	12,657 (36.3)	15.7 (15.0, 16.3)	6.1 (5.7, 6.5)	3.0 (2.7, 3.3)
*Missing*	2,880 (8.3)	10.0 (8.9, 11.1)	2.8 (2.2, 3.4)	2.2 (1.7, 2.8)
Mother’s marital or cohabitation status				
*Yes*	26,556 (76.3)	11.1 (10.7, 11.4)	4.5 (4.3, 4.8)	2.8 (2.6, 3.0)
*No*	8,365 (24.0)	10.9 (10.2, 11.5)	4.1 (3.7, 4.6)	2.6 (2.3, 3.0)
*Missing*	59 (0.2)	5.1 (0.0, 10.7)	1.7 (0.0, 5.0)	5.1 (0.0, 10.7)
Household wealth quintile				
*1, poorest*	10,531 (30.2)	16.2 (15.5, 16.9)	6.2 (5.7, 6.6)	2.6 (2.3, 2.9)
*2*	7,540 (21.6)	10.9 (10.2, 11.6)	4.8 (4.3, 5.3)	2.6 (2.3, 3.0)
*3*	6,638 (19.0)	8.6 (7.9, 9.2)	3.5 (3.0, 3.9)	3.0 (2.6, 3.5)
*4*	5,668 (16.3)	7.9 (7.2, 8.6)	3.4 (2.9, 3.8)	2.6 (2.2, 3.0)
*5, richest*	4,485 (12.9)	6.6 (5.9, 7.3)	2.6 (2.1, 3.1)	3.0 (2.5, 3.5)

CI – confidence interval

*Values are presented as weighted prevalence percentages with 95% confidence intervals in parentheses, accounting for complex survey design. Sample sizes (No.) are unweighted counts. For each factor, the ‘Missing’ row indicates the number and percentage of observations with missing data for that variable.

The distribution of malnutrition among relevant factors is presented in **Table 2**. Stunting was highest (12.6%) among children aged 12–23 months (95% CI = 11.8, 13.3) and relatively lower among those aged 0–11 months and 48–59 months. Boys had higher prevalence of stunting, underweight, and wasting than girls. The prevalence of stunting and underweight showed a clear decreasing trend with higher maternal education levels and higher household wealth. For example, among children whose mothers had primary education or no education, the stunting prevalence was 20.5% (95% CI = 17.7, 23.3), while among those whose mothers had higher education, it was 5.6% (95% CI = 5.1, 6.1). Mothers who had experienced child death had a higher prevalence of stunting (16.6%) among their living children (95% CI = 14.7, 18.4).

In multivariable logistic regression models adjusted for country fixed effects (**Table 3**), we assessed the associations between each factor and malnutrition outcomes. At the child level, compared to infants aged 0–11 months, children aged 12–23 months (OR = 1.55; 95% CI = 1.30, 1.84), 24–35 months (OR = 1.42; 95% CI = 1.20, 1.68), and 36–47 months (OR = 1.30; 95% CI = 1.09, 1.56) had significantly higher odds of stunting. In contrast, these age groups had lower odds of wasting compared to the reference group, *e.g.* 12–23 months (OR = 0.64; 95% CI = 0.47, 0.86). Girls had significantly lower odds than boys for all three forms of malnutrition (stunting OR = 0.85; 95% CI = 0.76, 0.94). Regarding recent illness, children with fever in the past two weeks had significantly higher odds of underweight (OR = 1.36; 95% CI = 1.13, 1.63) and wasting (OR = 1.45; 95% CI = 1.12, 1.88) compared to those without fever. However, diarrhoea was associated with lower odds of stunting (OR = 0.82; 95% CI = 0.71, 0.96), a finding contrary to expectations, suggesting possible effect modification or confounding, which was explored further in interaction analyses. Cough was not significantly associated with any form of malnutrition.

**Table 3:** Odds ratios and 95% confidence intervals for stunting, underweight, and wasting by selected factors among children aged 0–59 mo in eight countries or regions of Latin America and the Caribbean

Factor	Stunting		Underweight		Wasting	
	**OR (95% CI)**	***P*-value**	**OR (95% CI)**	***P*-value**	**OR (95% CI)**	***P*-value**
Children’s age						
*0–11 mo*	Ref		Ref		Ref	
*12–23 mo*	1.55 (1.30, 1.84)	<0.0001	1.09 (0.86, 1.39)	0.4683	0.64 (0.47, 0.86)	0.0034
*24–35 mo*	1.42 (1.20, 1.68)	<0.0001	1.17 (0.92, 1.48)	0.1935	0.58 (0.41, 0.81)	0.0012
*36–47 mo*	1.30 (1.09, 1.56)	0.0033	1.07 (0.84, 1.35)	0.5945	0.51 (0.38, 0.70)	<0.0001
*48–59 mo*	1.10 (0.91, 1.34)	0.3194	1.08 (0.83, 1.41)	0.5535	0.59 (0.44, 0.80)	0.0006
Children’s sex						
*Male*	Ref		Ref		Ref	
*Female*	0.85 (0.76, 0.94)	0.0025	0.81 (0.70, 0.94)	0.0050	0.76 (0.63, 0.92)	0.0057
Diarrhoea in past two weeks						
*No*	Ref		Ref		Ref	
*Yes*	0.82 (0.71, 0.96)	0.0123	0.93 (0.74, 1.19)	0.5741	0.92 (0.68, 1.23)	0.5644
Fever in past two weeks						
*No*	Ref		Ref		Ref	
*Yes*	1.08 (0.95, 1.24)	0.2512	1.36 (1.13, 1.63)	0.0010	1.45 (1.12, 1.88)	0.0047
Cough in past two weeks						
*No*	Ref		Ref		Ref	
*Yes*	0.92 (0.80, 1.04)	0.1869	1.05 (0.86, 1.28)	0.6095	0.97 (0.76, 1.26)	0.8385
Mother’s age						
*20–34 y*	Ref		Ref		Ref	
*15–19 y*	1.06 (0.86, 1.31)	0.5859	0.85 (0.63, 1.14)	0.2717	1.36 (0.94, 1.97)	0.1077
*35–49 y*	0.93 (0.80, 1.07)	0.2976	1.03 (0.85, 1.24)	0.7836	1.26 (0.99, 1.61)	0.0590
Mother’s education level						
*Primary or none*	Ref		Ref		Ref	
*Secondary*	0.79 (0.69, 0.91)	0.0009	0.72 (0.59, 0.88)	0.0014	0.89 (0.68, 1.18)	0.4288
*Higher*	0.62 (0.51, 0.75)	<0.0001	0.48 (0.35, 0.67)	<0.0001	0.77 (0.53, 1.12)	0.1677
Whether the mother has deceased children						
*No*	Ref		Ref		Ref	
*Yes*	1.46 (1.18, 1.81)	0.0006	1.42 (1.05, 1.92)	0.0221	1.11 (0.73, 1.71)	0.6245
Mother ever consumed alcohol						
*No*	Ref		Ref		Ref	
*Yes*	0.85 (0.75, 0.97)	0.0150	0.83 (0.69, 0.99)	0.0431	0.79 (0.63, 0.99)	0.0376
Mother’s marital or cohabitation status						
*No*	Ref		Ref		Ref	
*Yes*	0.99 (0.86, 1.13)	0.8481	0.90 (0.75, 1.08)	0.2542	0.76 (0.60, 0.95)	0.0182
Household wealth quintile						
*1, poorest*	Ref		Ref		Ref	
*2*	0.69 (0.59, 0.80)	<0.0001	0.82 (0.68, 0.99)	0.0379	1.10 (0.83, 1.47)	0.5048
*3*	0.55 (0.47, 0.64)	<0.0001	0.71 (0.57, 0.88)	0.0021	1.51 (1.13, 2.02)	0.0055
*4*	0.50 (0.41, 0.61)	<0.0001	0.65 (0.51, 0.82)	0.0003	1.16 (0.86, 1.57)	0.3223
*5, richest*	0.59 (0.47, 0.76)	<0.0001	0.73 (0.51, 1.04)	0.0775	1.44 (1.02, 2.04)	0.0404

CI – confidence interval, OR – odds ratio

Regarding maternal factors, compared to mothers with primary education or less, those with secondary education (OR = 0.79; 95% CI = 0.69, 0.91) or higher education (OR = 0.62; 95% CI = 0.51, 0.75) had significantly lower odds of stunting in their children, and a similar pattern was observed for underweight. In country-specific analyses, this association showed a consistent trend across countries (Figure S2 in the **Online Supplementary Document**). In addition, mothers who had experienced child death had significantly higher odds of stunting (OR = 1.46; 95% CI = 1.18, 1.81) and underweight (OR = 1.42; 95% CI = 1.05, 1.92) in their living children. Notably, mothers who reported ever consuming alcohol had lower odds of stunting (OR = 0.85; 95% CI = 0.75, 0.97), underweight (OR = 0.83; 95% CI = 0.69, 0.99), and wasting (OR = 0.79; 95% CI = 0.63, 0.99) in their children. Having a spouse or cohabiting partner was associated with lower odds of wasting (OR = 0.76; 95% CI = 0.60, 0.95). Furthermore, compared to the poorest households, higher household wealth was associated with significantly lower odds of stunting and underweight (*e.g*. richest *vs*. poorest quintile for stunting OR = 0.59; 95% CI = 0.47, 0.76). However, the pattern for wasting differed: children from middle-wealth households had higher odds of wasting than those from the poorest households (OR = 1.51; 95% CI = 1.13, 2.02). Country-specific forest plots further illustrated the patterns of association between household wealth and malnutrition across countries (Figure S2 in the **Online Supplementary Document**).

Given that stunting was common in the study population (prevalence >10%), we calculated PRs as a sensitivity analysis to enhance the robustness of our findings. To maintain consistency in the analytic approach, we also estimated PRs for underweight and wasting. The direction and statistical significance of the associations observed in the main logistic regression models for all three malnutrition outcomes remained consistent in the PR models (Table S4 in the **Online Supplementary Document**). Subsequently, subgroup analyses restricted to children aged 0–35 months (Tables S5 and S6 in the **Online Supplementary Document**) and 0–23 months (Tables S7 and S8 in the **Online Supplementary Document**) showed that the direction and magnitude of the main associations were generally similar to those in the main model. Among infants and children aged 0–23 months, never having been breastfed was associated with higher odds of underweight (OR = 0.50; 95% CI = 0.34, 0.74) (Table S8 in the **Online Supplementary Document**). Sensitivity analyses excluding Cuba (Tables S9 and S10 in the **Online Supplementary Document**) or excluding Turks and Caicos Islands due to its very small sample size (Table S11 in the **Online Supplementary Document**) showed that the effect estimates for the main findings remained stable, indicating that the main model results were not sensitive to data from specific countries. In the analysis excluding Cuba, child and maternal health insurance coverage variables were introduced, and maternal health insurance was associated with lower odds of wasting in children (OR = 0.74; 95% CI = 0.56, 0.97).

To further investigate the unexpected inverse association between diarrhoea and stunting observed in the main model, we tested for a possible interaction between diarrhoea and age group (Table S12 in the **Online Supplementary Document**). The analysis revealed a significant multiplicative interaction between diarrhoea and the 24–35 months age group (ROR = 1.93; 95% CI = 1.20, 3.10, *P* = 0.0070). This indicates that the association between diarrhoea and stunting differs across age groups: among children aged 24–35 months, a critical period often corresponding to weaning – the strength of the association between diarrhoea and stunting was nearly twice that observed in infants aged 0–11 months.

## DISCUSSION

This study analysed the prevalence and factors associated with three forms of childhood undernutrition: stunting, underweight, and wasting, among children under five years of age in eight countries of Latin America and the Caribbean. The findings indicate that the overall prevalence of childhood undernutrition in the region was generally consistent with recent regional estimates [9], yet significant disparities existed across population subgroups defined by demographic and socioeconomic characteristics, suggesting that public health challenges persist, particularly among socioeconomically disadvantaged groups.

This study found that maternal education and household wealth were the most robust factors associated with child nutritional status. Compared to children whose mothers had primary education or less, those whose mothers had secondary or higher education had significantly lower odds of stunting and underweight, a pattern that was consistent in direction across countries (Figure S2 in the **Online Supplementary Document**). This finding underscores the role of education as a fundamental social determinant, which may influence child nutrition both directly, through improved health literacy and care practices, and indirectly, by enhancing women’s autonomy within households and society [6,8]. From a policy perspective, this implies that investing in girls’ and women’s education is not only a development goal in itself but also a key strategy for improving intergenerational health. Similarly, the inverse association between household wealth and both stunting and underweight suggests that poverty reduction and social protection policies (*e.g*. cash transfer programmes, food subsidies) may positively influence child nutrition. However, the association between wealth and wasting was more complex: children from middle wealth households had higher odds of wasting compared to those from the poorest households. This seemingly paradoxical finding is consistent with some reports from Latin America and may reflect the ongoing ‘nutrition transition’ in the region [10,25]. As economies develop and urbanisation progresses, higher income groups may experience rapid dietary shifts (*e.g*. increased consumption of processed foods, erosion of traditional dietary patterns) without a corresponding improvement in health literacy and adaptive capacity, potentially leading to micronutrient deficiencies or wasting related to unhealthy diets. This suggests that nutrition interventions cannot simply target ‘income enhancement’ but require accompanying health education and food environment improvements to guide families towards healthier consumption choices.

This study found a significant association between child age and stunting, with the highest odds observed in the 12–35 months age group. This finding is highly consistent with the established understanding that the first 1000 days of life represent a critical window for growth – a period coinciding with complementary feeding introduction and weaning, and the highest vulnerability to growth faltering [3]. Our observation provides empirical support from the region for the importance of nutrition interventions during this window. More importantly, through interaction analysis, this study revealed a significant age-specific association between diarrhoea and stunting. In the unstratified main model, diarrhoea appeared to be inversely associated with stunting, a finding contrary to biological plausibility and inconsistent with most previous studies [26]. Further interaction analysis clarified this paradox: among children aged 24–35 months (the critical weaning period), the positive association between diarrhoea and stunting was nearly twice as strong as that observed in infants aged 0–11 months. This indicates that the ‘average effect’ in the main model masked substantial differences in the association across age groups. During infancy, the protective effect of breastfeeding may offset or confound the true impact of diarrhoea. During the weaning period, however, as the immune protection provided by breast milk gradually diminishes and children’s exposure to contaminated food and water increases, the negative impact of diarrhoea on nutritional status becomes apparent. This finding underscores that public health measures targeting diarrhoea, such as promoting oral rehydration salts, hand hygiene, and safe water should specifically focus on weaning-age children and their caregivers.

In the subgroup analysis of infants and children aged 0–23 months, this study found that ever having been breastfed was significantly associated with lower odds of underweight, a direction of effect consistent with expectations. This finding further supports the global recommendation of exclusive breastfeeding for the first six months and continued breastfeeding up to two years of age or beyond [15]. Additionally, in sensitivity analyses excluding Cuba, maternal health insurance coverage was associated with lower odds of wasting in children. This finding supports the necessity of integrating nutrition services into routine maternal and child health packages through universal health coverage or targeted health insurance schemes [27].

This study observed an association between maternal report of ever consuming alcohol and lower odds of child undernutrition. This finding appears counterintuitive, as prenatal alcohol consumption is well-established to be associated with foetal alcohol spectrum disorders and adverse birth outcomes [28]. However, this study measured ‘ever consumed alcohol’ in a general sense, not specifically alcohol use during pregnancy; therefore, a direct biological mechanism is unclear. A more plausible explanation is that this association reflects residual socioeconomic confounding. In some Latin American countries, alcohol consumption may be correlated with higher socioeconomic status, urban residence, or modern lifestyles, factors which themselves are protective against child undernutrition [6]. Conversely, mothers who never drank may be more likely to belong to religiously or culturally conservative groups, which may also be associated with lower socioeconomic status [29]. The association lacked a dose-response relationship and persisted after adjusting for education, wealth, and other covariates, suggesting the presence of deeper, unmeasured confounding factors (*e.g*. social capital, religious beliefs, community environment). Therefore, we interpret this finding with great caution: it should not be misinterpreted as ‘alcohol being beneficial for child health’ but rather serves as a cautionary example of how observed associations in cross-sectional studies may reflect complex sociocultural patterns rather than direct biological effects. This also highlights the importance of understanding the sociocultural context of behaviours and exercising caution when interpreting counterintuitive findings in public health research.

This study leveraged large-scale, standardised, and population-representative multi-country survey data. The analysis rigorously accounted for the complex survey design and partially controlled for unmeasured country-level confounding by including country fixed effects. Furthermore, we conducted PR analyses to further verify the robustness of our findings and performed subgroup and interaction analyses. These methodological efforts enhance the reliability of our conclusions and provide more empirically grounded scientific evidence for regional public health policy.

However, the limitations of this study must be fully acknowledged. First, the cross-sectional design precludes causal inference; we can only report statistical associations, not causal relationships. Second, while we selected variables based on prior research and the UNICEF conceptual framework on maternal and child nutrition, several important dimensions of this framework could not be included due to limitations in the availability and comparability of core indicators across the multi-country survey data. Specifically, variables such as dietary diversity, maternal nutritional status (*e.g*. maternal height, body mass index), birth outcomes (*e.g*. birth weight, gestational age), and household food security were not collected consistently across all participating MICS6 countries or could not be harmonised cross-nationally due to differences in questionnaire modules. These unmeasured factors may contribute to residual confounding. Third, most exposure variables in this study were based on self-report, which may be subject to recall bias or reporting bias due to social desirability, potentially affecting the accuracy of the results. Fourth, for missing data, we employed multiple imputation by chained equations. The imputation model for the main analysis included all three outcome indicators, 12 core variables, rescaled sampling weights, and country indicators to support the missing at random assumption. However, to avoid non-convergence due to high dimensionality, the imputation model did not include PSU and stratification variables, which may affect imputation precision. Fifth, although we included country fixed effects in our models to control for time-invariant confounding at the country level, this approach cannot capture heterogeneity in effect sizes across countries, nor can it correct for bias arising from differences in survey implementation, questionnaire wording, or measurement error across countries. The sample size for some countries (*e.g*. Turks and Caicos Islands) was too small to permit reliable country-stratified analyses. While we presented country-stratified forest plots in Figure S2 in the **Online Supplementary Document**, the precision of estimates for some countries is limited by sample size; these should be interpreted in terms of directional trends rather than precise point estimates.

## CONCLUSIONS

Childhood undernutrition in Latin America and the Caribbean remains a significant public health challenge, deeply rooted in structural socioeconomic inequalities. The findings of this study provide empirical evidence to inform relevant policy formulation:

(1) continued investment in girls’ education and women’s empowerment

(2) targeting nutrition interventions to the first 1000 days of life, particularly infection control and feeding support for weaning-age children

(3) accompanying poverty reduction and social protection policies with health education and food environment improvements to address challenges arising from the nutrition transition

(4) integrating nutrition services into universal health coverage or maternal and child health insurance schemes.

Future research should employ longitudinal designs or quasi-experimental methods to further elucidate the causal pathways between key factors and child nutritional status. Concurrently, countries are encouraged to strengthen their data systems, improve the comparability of survey instruments and the completeness of core variables, to more comprehensively capture the multidimensional determinants within the UNICEF framework and provide a more robust foundation for regional and global nutrition monitoring.

**Data availability:** The data used in this study are publicly accessible and were obtained from the Multiple Indicator Cluster Surveys (MICS6) conducted in eight countries/regions across Latin America and the Caribbean. These datasets can be requested by registered users through the UNICEF MICS website (https://mics.unicef.org/surveys).

## Additional material


Online Supplementary Document
