# Supplementary material for: Factors associated with child stunting, underweight, and wasting in eight Latin American and Caribbean countries and regions: a regional analysis using Multiple Indicator Cluster Surveys
Source: J Glob Health. 2026 Jul 17;16:04201. doi: 10.7189/jogh.16.04201 (PMC13377585; doi:10.7189/jogh.16.04201)
Supplement: Online Supplementary Document [file jogh-16-04201-s001.pdf]

Supplement to: Zhang X, Deng Y, Xie T, Gao S, Tao Z, Teng F, Hu G, Wang F, Wang X, Zhao G. Factors associated with child stunting, underweight, and wasting in eight Latin American and Caribbean countries and regions: a regional analysis using Multiple Indicator Cluster Surveys. J Glob Health. 2026;16:04201.

Correspondence to:

Xiaohui Wang

School of Public Health, Lanzhou University

Lanzhou 730000, China

China

wangxiaohui@lzu.edu.cn

Guanlan Zhao

School of Public Health, Lanzhou University

Lanzhou 730000, China

China

zhaogl@lzu.edu.cn

Content

Checklist S1. STROBE Statement — Checklist of items that should be included in reports of cross-sectional studies........1

Table S1. Availability, target populations, and missingness mechanisms of all selected covariates.... 5

Table S2. Variance Inflation Factors (VIF) for all predictors in multivariable models of stunting, underweight, and wasting........ 7

Table S3. Prevalence of Stunting, Underweight, and Wasting Among Children Aged 0–59 Months in Eight Countries or Regions of Latin America and the Caribbean........8

Table S4. Prevalence Ratios (PR) and 95% Confidence Intervals (CI) for Stunting, Underweight, and Wasting by Selected Factors Among Children Aged 0–59 Months in Eight Countries or Regions of Latin America and the Caribbean........9

Table S5. Prevalence of Stunting, Underweight, and Wasting by Selected Factors Among Children Aged 0–35 Months in Eight Countries or Regions of Latin America and the Caribbean........ 11

Table S6. Odds Ratios (OR) and 95% Confidence Intervals (CI) for Stunting, Underweight, and Wasting by Selected Factors Among Children Aged 0–35 Months in Eight Countries or Regions of Latin America and the Caribbean........14

Table S7. Prevalence of Stunting, Underweight, and Wasting by Selected Factors Among Children Aged 0–23 Months in Eight Countries or Regions of Latin America and the Caribbean........ 16

Table S8. Odds Ratios (OR) and 95% Confidence Intervals (CI) for Stunting, Underweight, and Wasting by Selected Factors Among Children Aged 0–23 Months in Eight Countries or Regions of Latin America and the Caribbean........19

Table S9. Prevalence of Stunting, Underweight, and Wasting by Selected Factors Among Children Aged 0–59 Months in Seven Countries or Regions of Latin America and the Caribbean Excluding Cuba........22

Table S10. Odds Ratios (OR) and 95% Confidence Intervals (CI) for Stunting, Underweight, and Wasting by Selected Factors Among Children Aged 0–59 Months in Seven Countries or Regions of Latin America and the Caribbean Excluding Cuba........25

Table S11. Odds Ratios (OR) and 95% Confidence Intervals (CI) for Stunting, Underweight, and Wasting by Selected Factors Among Children Aged 0–59 Months in Seven Countries or Regions of Latin America and the Caribbean Excluding Turks and Caicos Islands........27

Table S12. Ratio of Odds Ratios (ROR) and 95% Confidence Intervals (CI) for the Interactions of Diarrhea by Age on Stunting and Household Wealth by Country on Wasting........29

Figure S1. Diagnostic trace plots for convergence of the multiple imputation by chained equations (MICE) procedure........ 30

Figure S2. Odds Ratios (OR) and 95% Confidence Intervals (CI) for Stunting, Underweight, and Wasting by Maternal Education Level and Household Wealth Quintile Among Children Aged 0–59 Months in Seven Countries or Regions of Latin America and the Caribbean Excluding Turks and Caicos Islands........32

Checklist S1. STROBE Statement—Checklist of items that should be included in reports of cross-sectional studies

	Item Description	Location (or reason for not reporting)
**Title and abstract**		
1a. Indicate the study's design	Indicate the study's design with a commonly used term in the title or the abstract.	Title; Abstract, para 2
1b. Abstract	Provide in the abstract an informative and balanced summary of what was done and what was found.	Abstract, all paragraphs
**Introduction**		
2. Background / rationale	Explain the scientific background and rationale for the investigation being reported.	Introduction, paras 1–3
3. Objectives	State specific objectives, including any prespecified hypotheses.	Introduction, final para
**Methods**		
4. Study design	Present key elements of study design early in the paper.	Methods, para 1 (Study design and data source)
5. Setting	Describe the setting, locations, and relevant dates, including periods of recruitment, exposure, follow-up, and data collection.	Methods (Study design and data source), para 1
6a. Eligibility criteria	**Cohort study:** Give the eligibility criteria, and the sources and methods of selection of participants. Describe methods of follow-up. **Case-control study:** Give the eligibility criteria, and the sources and methods of case ascertainment and control selection. Give the rationale for the choice of cases and controls. **Cross-sectional study:** Give the eligibility criteria, and the sources and methods of selection of participants.	Methods (Study population and sampling size), paras 1–3; Figure 1
6b. Matching criteria	**Cohort study:** For matched studies, give matching criteria and number of exposed and unexposed. **Case-control study:** For matched studies, give matching criteria and the number of controls per case.	Not applicable
7. Variables	Clearly define all outcomes, exposures, predictors, potential confounders, and effect modifiers. Give diagnostic criteria, if applicable.	Methods (Outcomes), para 1; Methods (Exposures), paras 1–3; Table 1

8. Data sources / measurement	For each variable of interest give sources of data and details of methods of assessment (measurement). Describe comparability of assessment methods if there is more than one group.	Methods (Exposures), paras 1 and 3
9. Bias	Describe any efforts to address potential sources of bias.	Methods (Statistical analysis), paras 2–3; Discussion (Limitations), para 1
10. Study size	Explain how the study size was arrived at.	Methods (Study population and sampling size), paras 2–3; Figure 1
11. Quantitative variables	Explain how quantitative variables were handled in the analyses. If applicable, describe which groupings were chosen, and why.	Methods (Exposures), para 4
12a. Statistical methods	Describe all statistical methods, including those used to control for confounding.	Methods (Statistical analysis), paras 2–5
12b. Statistical methods – subgroups and interactions	Describe any methods used to examine subgroups and interactions.	Methods (Statistical analysis), para 6
12c. Statistical methods – missing data	Explain how missing data were addressed.	Methods (Statistical analysis), para 3
12di. Statistical methods – loss to follow-up	**Cohort study:** If applicable, describe how loss to follow-up was addressed.	Not applicable
12dii. Statistical methods – matching cases and controls	**Case-control study:** If applicable, explain how matching of cases and controls was addressed.	Not applicable
12diii. Statistical methods – sampling strategy	**Cross-sectional study:** If applicable, describe analytical methods taking account of sampling strategy.	Methods (Statistical analysis), para 2
12e. Statistical methods – sensitivity analyses	Describe any sensitivity analyses.	Methods (Statistical analysis), para 6
**Results**		
13a. Participant numbers	Report the numbers of individuals at each stage of the study—e.g., numbers potentially eligible,	Results, para 1; Methods (Study population and

	examined for eligibility, confirmed eligible, included in the study, completing follow-up, and analysed; Consider use of a flow diagram.	sampling size), para 3; Figure 1
13b. Participants – non-participation	Give reasons for non-participation at each stage.	Methods (Study population and sampling size), para 3; Figure 1
13c. Participants – flow diagram	Consider use of a flow diagram.	Figure 1
14a. Descriptive data – participant characteristics	Give characteristics of study participants (e.g., demographic, clinical, social) and information on exposures and potential confounders. Present the information in a table.	Results, para 1; Table 2
14b. Descriptive data – missing data	Indicate the number of participants with missing data for each variable of interest.	Table 2 (Missing rows for each variable)
14c. Descriptive data – follow-up time	**Cohort study:** Summarise follow-up time—e.g., average and total amount.	Not applicable
15. Outcome data	**Cohort study:** Report numbers of outcome events or summary measures over time. **Case-control study:** Report numbers in each exposure category, or summary measures of exposure. **Cross-sectional study:** Report numbers of outcome events or summary measures.	Results, para 1; Table 2
16a. Main results	Give unadjusted estimates and, if applicable, confounder-adjusted estimates and their precision (e.g., 95% confidence intervals). Make clear which confounders were adjusted for and why they were included.	Table 3; Methods (Exposures), paras 1–2; Methods (Statistical analysis), para 5
16b. Main results – category boundaries	Report category boundaries when continuous variables were categorised.	Table 3 (child age categories)
16c. Main results – risk	If relevant, consider translating estimates of relative risk into absolute risk for a meaningful time period.	Not applicable
17. Other analyses	Report other analyses done—e.g., analyses of subgroups and interactions, and sensitivity analyses.	Results, paras 4–5; Tables S4–S12; Figure S2
**Discussion**		
18. Key results	Summarise key results with reference to study objectives.	Discussion, para 1

19. Limitations	Discuss limitations of the study, taking into account sources of potential bias or imprecision. Discuss both direction and magnitude of any potential bias.	Discussion, para 7 (Limitations)
20. Interpretation	Give a cautious overall interpretation considering objectives, limitations, multiplicity of analyses, results from similar studies, and other relevant evidence.	Discussion, paras 3–6
21. Generalisability	Discuss the generalisability (external validity) of the study results.	Discussion, para 7 (final sentence)
**Other information**		
22. Funding	Give the source of funding and the role of the funders for the present study and, if applicable, for the original study on which the present article is based.	Acknowledgements; Funding

Table S1. Availability, target populations, and missingness mechanisms of all selected covariates

Factor	Target Population (Age Group)	Country Availability	Overall Missingness	Principal Missingness Mechanism & Handling Strategy
Panel A: Core Variables				
Children's age	All children (0–59 months)	All countries	0.0%	Complete data.
Children's sex	All children (0–59 months)	All countries	0.0%	Complete data.
Household wealth quintile	All children (0–59 months)	All countries	0.0%	Complete data.
Mother's age	All children (0–59 months)	All countries	0.0%	Complete data.
Mother's education level	All children (0–59 months)	All countries	2.3%	True item non-response (Missing at Random). Handled via MICE.
Mother's marital or cohabitation status	All children (0–59 months)	All countries	0.2%	True item non-response. Handled via MICE.
Mother ever consumed alcohol	All children (0–59 months)	All countries	8.3%	True item non-response. Handled via MICE.
Whether the mother has deceased children	All children (0–59 months)	All countries	< 0.1%	True item non-response. Handled via MICE.
Diarrhea in past 2 weeks	All children (0–59 months)	All countries	0.2%	True item non-response. Handled via MICE.
Fever in past 2 weeks	All children (0–59 months)	All countries	0.2%	True item non-response. Handled via MICE.
Cough in past 2 weeks	All children (0–59 months)	All countries	0.2%	True item non-response. Handled via MICE.
Panel B: Age-Specific & Structurally Missing Variables				
Ever breastfed	0–35 months	All countries	42.0%	Structural missingness: Questionnaire module restricted to children under 3 years of age. Analyzed separately in the 0–35 months subgroup.
Safe disposal of child's stool	0–35 months	All countries	42.0%	Structural missingness: Module administered predominantly for children under 3 years. Analyzed separately in the 0–35 months subgroup.
Cesarean delivery	0–23 months	All countries	52.5%	Structural missingness: MICS maternal health module restricted to the last live birth within the past 2 years. Analyzed in the 0–23 months subgroup.
Received antenatal care	0–23 months	All countries	50.3%	Structural missingness: Same as Cesarean delivery. Analyzed separately in the 0–23 months subgroup.

Breastfeeding guidance after delivery	0–23 months	All countries	50.4%	Structural missingness: Same as Cesarean delivery. Analyzed separately in the 0–23 months subgroup.
Panel C: Variables with Country-Specific Unavailability				
Child health insurance coverage	All children (0–59 months)	Missing in Cuba	0.1%	Country-level structural missingness: Not collected in the Cuba survey. Excluded from pooled main analysis to avoid misclassification bias; evaluated in a 7-country sensitivity model.
Mother's health insurance coverage	All children (0–59 months)	Missing in Cuba	0.1%	Country-level structural missingness: Same as child health insurance. Evaluated in a 7-country sensitivity model.

Note: Missingness percentage for insurance variables is calculated among the 7 countries where the question was administered. MICE, Multiple Imputation by Chained Equations. Missingness in Panel B variables among older children is structural (driven by questionnaire skip patterns) rather than random non-response. To prevent imputation bias, these variables were excluded from the main pooled models and analyzed within appropriate age-restricted subgroups.

Table S2. Variance Inflation Factors (VIF) for all predictors in multivariable models of stunting, underweight, and wasting

Factor	Df	Stunting		Underweight		Wasting	
		Max GVIF	Max GVIF$^{\wedge(1/(2*Df))}$	Max GVIF	Max GVIF$^{\wedge(1/(2*Df))}$	Max GVIF	Max GVIF$^{\wedge(1/(2*Df))}$
Children's age	4	1.08	1.01	1.08	1.01	1.09	1.01
Children's sex	1	1.00	1.00	1.00	1.00	1.00	1.00
Diarrhea in past 2 weeks	1	1.06	1.03	1.06	1.03	1.07	1.04
Fever in past 2 weeks	1	1.27	1.13	1.30	1.14	1.30	1.14
Cough in past 2 weeks	1	1.25	1.12	1.28	1.13	1.28	1.13
Mother's age	2	1.14	1.03	1.14	1.03	1.17	1.04
Mother's education level	2	2.04	1.20	2.02	1.19	1.86	1.17
Whether the mother has deceased children	1	1.03	1.02	1.04	1.02	1.05	1.02
Mother ever consumed alcohol	1	1.29	1.14	1.31	1.15	1.19	1.09
Mother's marital or cohabitation status	1	1.04	1.02	1.04	1.02	1.04	1.02
Household wealth quintile	4	1.31	1.03	1.31	1.03	1.32	1.04
Country or regions	7	2.11	1.05	2.10	1.05	1.89	1.05

Note: Maximum generalized variance inflation factors (GVIF) and their adjusted values (GVIF$^{\wedge(1/(2*Df))}$) across 20 multiple imputed datasets are presented for each variable in the main multivariable models. values below 2 indicate that no significant multicollinearity influenced the model results.

Table S3. Prevalence of Stunting, Underweight, and Wasting Among Children Aged 0-59 Months in Eight Countries or Regions of Latin America and the Caribbean

Countries or Regions	Year of survey	Sample size	Prevalence, % (95% CI)		
			Stunting	Underweight	Wasting
Argentina	2019-2020	5, 481	11.1 (10.3-12.0)	2.6 (2.2-3.1)	2.2 (1.9-2.7)
Dominican Republic	2019	7, 697	7.2 (6.6-7.8)	3.4 (3.0-3.8)	2.3 (2.0-2.7)
Costa Rica	2018	3, 051	7.8 (6.9-8.8)	3.2 (2.6-3.8)	2.0 (1.5-2.5)
Cuba	2019	5, 094	6.9 (6.2-7.6)	2.2 (1.8-2.6)	2.5 (2.1-2.9)
Guyana	2019-2020	2, 421	11.9 (10.6-13.2)	7.3 (6.3-8.3)	5.9 (5.0-6.9)
Honduras	2019	7, 765	20.3 (19.4-21.2)	7.2 (6.6-7.8)	1.8 (1.5-2.1)
Suriname	2018	3, 110	6.9 (6.0-7.8)	6.3 (5.5-7.2)	5.9 (5.1-6.8)
Turks and Caicos Islands	2019-2020	243	2.1 (0.3-3.9)	0.4 (0.0-1.2)	0.8 (0.0-2.0)

Note: CI, confidence interval.

Table S4. Prevalence Ratios (PR) and 95% Confidence Intervals (CI) for Stunting, Underweight, and Wasting by Selected Factors Among Children Aged 0-59 Months in Eight Countries or Regions of Latin America and the Caribbean

Factor	Stunting		Underweight		Wasting	
	PR (95% CI)	*P*-value	PR (95% CI)	*P*-value	PR (95% CI)	*P*-value
Children's age						
0-11 months	Ref		Ref		Ref	
12-23 months	1.45 (1.25-1.69)	<0.0001	1.09 (0.87-1.36)	0.4664	0.65 (0.49-0.87)	0.0033
24-35 months	1.35 (1.17-1.57)	<0.0001	1.16 (0.93-1.44)	0.1945	0.59 (0.43-0.81)	0.0012
36-47 months	1.26 (1.08-1.47)	0.0035	1.06 (0.85-1.33)	0.5932	0.53 (0.39-0.71)	<0.0001
48-59 months	1.09 (0.92-1.29)	0.3228	1.08 (0.84-1.38)	0.5533	0.60 (0.45-0.81)	0.0006
Children's sex						
Boy	Ref		Ref		Ref	
Girl	0.87 (0.79-0.95)	0.0026	0.82 (0.71-0.94)	0.0051	0.77 (0.64-0.93)	0.0057
Diarrhea in past 2 weeks						
No	Ref		Ref		Ref	
Yes	0.85 (0.75-0.97)	0.0135	0.94 (0.75-1.17)	0.5729	0.92 (0.69-1.22)	0.5654
Fever in past 2 weeks						
No	Ref		Ref		Ref	
Yes	1.07 (0.95-1.20)	0.2421	1.33 (1.12-1.58)	0.0009	1.43 (1.12-1.82)	0.0046
Cough in past 2 weeks						
No	Ref		Ref		Ref	
Yes	0.93 (0.83-1.03)	0.1725	1.05 (0.87-1.26)	0.6123	0.98 (0.76-1.24)	0.8395
Mother's age						
20-34 years	Ref		Ref		Ref	
15-19 years	1.05 (0.88-1.25)	0.5879	0.85 (0.65-1.13)	0.2731	1.34 (0.94-1.91)	0.1059
35-49 years	0.94 (0.83-1.06)	0.2897	1.02 (0.86-1.22)	0.7966	1.25 (0.99-1.58)	0.0582

Mother's education level						
Primary or none	Ref		Ref		Ref	
Secondary	0.82 (0.73-0.92)	0.0012	0.74 (0.61-0.89)	0.0014	0.90 (0.69-1.17)	0.4250
Higher	0.65 (0.54-0.78)	<0.0001	0.50 (0.36-0.68)	<0.0001	0.78 (0.55-1.11)	0.1655
Whether the mother has deceased children						
No	Ref		Ref		Ref	
Yes	1.36 (1.14-1.61)	0.0004	1.38 (1.05-1.81)	0.0210	1.11 (0.74-1.67)	0.6258
Mother ever consumed alcohol						
No	Ref		Ref		Ref	
Yes	0.87 (0.78-0.98)	0.0191	0.84 (0.70-1.00)	0.0439	0.79 (0.64-0.99)	0.0370
Mother's marital or cohabitation status						
No	Ref		Ref		Ref	
Yes	0.99 (0.88-1.11)	0.8153	0.90 (0.76-1.08)	0.2530	0.77 (0.62-0.95)	0.0176
Household wealth quintile						
1, poorest	Ref		Ref		Ref	
2	0.73 (0.65-0.83)	<0.0001	0.83 (0.70-0.99)	0.0397	1.10 (0.83-1.45)	0.5039
3	0.60 (0.52-0.69)	<0.0001	0.72 (0.59-0.89)	0.0023	1.48 (1.12-1.96)	0.0053
4	0.55 (0.46-0.65)	<0.0001	0.67 (0.53-0.83)	0.0003	1.16 (0.87-1.54)	0.3220
5, richest	0.64 (0.52-0.80)	0.0001	0.75 (0.53-1.04)	0.0835	1.42 (1.02-1.98)	0.0394

Note: PR, prevalence ratio; CI, confidence interval.

Table S5. Prevalence of Stunting, Underweight, and Wasting by Selected Factors Among Children Aged 0–35 Months in Eight Countries or Regions of Latin America and the Caribbean

Factor	Children observed, No. (%)	Prevalence, %(95%CI)		
		Stunting	Underweight	Wasting
Total	20,297 (100.0)	11.1 (10.4-11.8)	4.4 (4.0-4.8)	3.0 (2.6-3.3)
Children's age				
0-11 months	6,289 (31.0)	8.7 (7.7-9.8)	4.0 (3.3-4.7)	4.0 (3.2-4.8)
12-23 months	7,106 (35.0)	12.6 (11.4-13.8)	4.4 (3.7-5.1)	2.6 (2.1-3.1)
24-35 months	6,902 (34.0)	11.9 (10.8-13.0)	4.8 (4.1-5.5)	2.4 (1.8-2.9)
Children's sex				
Girl	9,930 (48.9)	9.2 (8.4-10.1)	3.7 (3.1-4.2)	2.4 (2.0-2.9)
Boy	10,367 (51.1)	13.0 (12.0-13.9)	5.2 (4.5-5.8)	3.5 (3.0-4.0)
Diarrhea in past two weeks				
Yes	2,932 (14.4)	10.3 (8.8-11.7)	4.7 (3.6-5.8)	2.6 (1.8-3.4)
No	17,312 (85.3)	11.2 (10.5-12.0)	4.4 (3.9-4.8)	3.0 (2.6-3.4)
Missing	53 (0.3)	18.8 (6.2-31.3)	8.9 (0.0-18.0)	1.2 (0.0-3.7)
Fever in past two weeks				
Yes	4,071 (20.1)	12.2 (10.8-13.6)	6.0 (5.0-7.0)	3.6 (2.8-4.5)
No	16,182 (79.7)	10.8 (10.1-11.6)	4.0 (3.6-4.5)	2.8 (2.4-3.2)
Missing	44 (0.2)	18.8 (0.0-40.2)	13.7 (0.0-35.2)	0.0 (0.0-0.0)
Cough in past two weeks				
Yes	4,798 (23.6)	11.4 (10.1-12.6)	5.7 (4.7-6.7)	3.2 (2.5-3.9)
No	15,462 (76.2)	11.0 (10.2-11.8)	4.0 (3.6-4.5)	2.9 (2.5-3.3)
Missing	37 (0.2)	17.5 (0.0-35.5)	5.9 (0.0-17.2)	1.5 (0.0-4.4)
Mother's age				

15-19 years	1,981 (9.8)	13.2 (10.9-15.6)	4.3 (3.1-5.6)	4.1 (2.8-5.5)
20-34 years	14,775 (72.8)	10.8 (10.0-11.5)	4.3 (3.8-4.8)	2.7 (2.3-3.1)
35-49 years	3,541 (17.4)	11.4 (9.9-12.9)	4.9 (3.9-5.9)	3.5 (2.6-4.5)
Mother’s education level				
Primary or none	5,533 (27.3)	16.3 (15.0-17.6)	6.8 (5.9-7.6)	2.9 (2.3-3.5)
Secondary	10,201 (50.3)	9.7 (8.8-10.7)	4.0 (3.4-4.6)	3.2 (2.6-3.7)
Higher	4,130 (20.3)	7.6 (6.1-9.1)	2.1 (1.4-2.7)	2.5 (1.8-3.3)
Missing	433 (2.1)	16.6 (11.5-21.8)	9.1 (4.8-13.5)	3.7 (1.8-5.7)
Mother's marital or cohabitation status				
Yes	15,505 (76.4)	10.9 (10.2-11.7)	4.4 (3.9-4.8)	2.9 (2.5-3.3)
No	4,757 (23.4)	11.8 (10.3-13.2)	4.6 (3.7-5.5)	3.1 (2.3-3.9)
Missing	35 (0.2)	9.0 (0.0-22.8)	7.2 (0.0-20.8)	2.9 (0.0-7.3)
Mother ever consumed alcohol				
Yes	11,579 (57.0)	9.3 (8.5-10.2)	3.7 (3.2-4.2)	2.8 (2.3-3.2)
No	7,749 (38.2)	14.0 (12.9-15.1)	5.7 (5.0-6.5)	3.4 (2.7-4.0)
Missing	969 (4.8)	11.8 (8.6-15.0)	3.5 (1.5-5.5)	2.2 (0.7-3.8)
Experienced child death				
Yes	831 (4.1)	16.5 (12.8-20.1)	8.8 (5.8-11.8)	3.7 (1.9-5.5)
No	19,460 (95.9)	10.9 (10.2-11.6)	4.2 (3.8-4.7)	2.9 (2.6-3.3)
Missing	6 (0.0)	37.4 (0.0-74.7)	0.0 (0.0-0.0)	0.0 (0.0-0.0)
Household wealth quintile				
1, poorest	6,301 (31.0)	15.1 (13.7-16.5)	6.1 (5.3-7.0)	3.0 (2.3-3.6)
2	4,391 (21.6)	12.0 (10.5-13.5)	4.9 (3.9-5.8)	2.6 (1.8-3.3)
3	3,830 (18.9)	9.4 (8.0-10.7)	3.5 (2.7-4.3)	3.5 (2.6-4.3)
4	3,235 (15.9)	8.2 (6.7-9.6)	3.6 (2.7-4.4)	2.7 (1.9-3.5)

5, richest	2,540 (12.5)	8.2 (6.5-9.8)	2.8 (1.8-3.9)	3.4 (2.3-4.4)
Ever breastfed				
Yes	18,866 (92.9)	10.9 (10.2-11.6)	4.2 (3.8-4.6)	2.9 (2.6-3.3)
No	1,357 (6.7)	13.7 (11.1-16.2)	7.2 (5.1-9.2)	3.5 (2.2-4.8)
Missing	74 (0.4)	12.0 (3.4-20.5)	4.7 (0.0-10.1)	0.3 (0.0-1.0)
Safe disposal of child's stool				
Yes	7,127 (35.1)	11.1 (10.0-12.3)	4.1 (3.4-4.8)	2.5 (1.9-3.0)
No	13,089 (64.5)	11.1 (10.3-11.9)	4.6 (4.1-5.1)	3.2 (2.8-3.7)
Missing	81 (0.4)	12.5 (4.3-20.7)	4.9 (0.0-10.2)	2.6 (0.0-5.9)

Note: CI, confidence interval.

Table S6. Odds Ratios (OR) and 95% Confidence Intervals (CI) for Stunting, Underweight, and Wasting by Selected Factors Among Children Aged 0-35 Months in Eight Countries or Regions of Latin America and the Caribbean

Factor	Stunting		Underweight		Wasting	
	OR (95% CI)	*P*-value	OR (95% CI)	*P*-value	OR (95% CI)	*P*-value
Children's age						
0-11 months	Ref		Ref		Ref	
12-23 months	1.51 (1.27-1.80)	<0.0001	1.08 (0.85-1.37)	0.5309	0.64 (0.47-0.87)	0.0043
24-35 months	1.37 (1.15-1.63)	0.0004	1.15 (0.90-1.48)	0.2636	0.58 (0.40-0.84)	0.0043
Children's sex						
Boy	Ref		Ref		Ref	
Girl	0.66 (0.58-0.76)	<0.0001	0.69 (0.57-0.84)	0.0002	0.69 (0.54-0.89)	0.0041
Diarrhea in past 2 weeks						
No	Ref		Ref		Ref	
Yes	0.80 (0.67-0.96)	0.0151	0.90 (0.68-1.19)	0.4641	0.82 (0.58-1.16)	0.2666
Fever in past 2 weeks						
No	Ref		Ref		Ref	
Yes	1.14 (0.96-1.34)	0.1362	1.23 (0.96-1.56)	0.0954	1.31 (0.93-1.85)	0.1213
Cough in past 2 weeks						
No	Ref		Ref		Ref	
Yes	0.97 (0.82-1.14)	0.6837	1.28 (0.99-1.65)	0.0554	1.09 (0.79-1.50)	0.6053
Mother's age						
20-34 years	Ref		Ref		Ref	
15-19 years	1.14 (0.91-1.44)	0.2570	0.85 (0.61-1.19)	0.3497	1.50 (1.01-2.23)	0.0449
35-49 years	0.98 (0.83-1.17)	0.8337	1.06 (0.82-1.36)	0.6709	1.31 (0.94-1.81)	0.1089
Mother's education level						
Primary or none	Ref		Ref		Ref	

Secondary	0.81 (0.69-0.94)	0.0078	0.76 (0.60-0.96)	0.0220	0.82 (0.59-1.15)	0.2609
Higher	0.75 (0.59-0.95)	0.0191	0.50 (0.33-0.75)	0.0009	0.74 (0.47-1.18)	0.2113
Mother's marital or cohabitation status						
No	Ref		Ref		Ref	
Yes	0.94 (0.80-1.11)	0.4889	0.84 (0.66-1.07)	0.1652	0.80 (0.60-1.07)	0.1337
Mother ever consumed alcohol						
No	Ref		Ref		Ref	
Yes	0.86 (0.74-1.00)	0.0436	0.78 (0.62-0.97)	0.0276	0.75 (0.56-1.00)	0.0536
Experienced child death						
No	Ref		Ref		Ref	
Yes	1.40 (1.06-1.86)	0.0176	1.66 (1.11-2.49)	0.0140	1.24 (0.73-2.10)	0.4349
Household wealth quintile						
1, poorest	Ref		Ref		Ref	
2	0.81 (0.68-0.97)	0.0196	0.88 (0.69-1.13)	0.3133	0.96 (0.66-1.40)	0.8242
3	0.63 (0.52-0.76)	<0.0001	0.67 (0.50-0.89)	0.0051	1.39 (0.96-2.00)	0.0776
4	0.56 (0.45-0.70)	<0.0001	0.71 (0.53-0.95)	0.0214	1.13 (0.77-1.64)	0.5368
5, richest	0.62 (0.47-0.80)	0.0004	0.67 (0.42-1.06)	0.0836	1.46 (0.95-2.26)	0.0838
Ever breastfed						
No	Ref		Ref		Ref	
Yes	0.81 (0.64-1.02)	0.0698	0.65 (0.48-0.90)	0.0083	0.93 (0.63-1.39)	0.7376
Safe disposal of child's stool						
No	Ref		Ref		Ref	
Yes	1.04 (0.90-1.19)	0.6352	0.95 (0.75-1.20)	0.6518	0.97 (0.68-1.39)	0.8881

Note: OR, odds ratio; CI, confidence interval.

Table S7. Prevalence of Stunting, Underweight, and Wasting by Selected Factors Among Children Aged 0–23 Months in Eight Countries or Regions of Latin America and the Caribbean

Factor	Children observed, No. (%)	Prevalence, %(95%CI)		
		Stunting	Underweight	Wasting
Total	13,395 (100.0)	10.8 (10.0-11.6)	4.3 (3.8-4.8)	3.3 (2.8-3.7)
Children's age				
0-11 months	6,289 (47.0)	8.8 (7.7-9.8)	4.1 (3.4-4.7)	4.0 (3.2-4.8)
12-23 months	7,106 (53.0)	12.7 (11.5-13.9)	4.5 (3.8-5.2)	2.6 (2.1-3.1)
Children's sex				
Girl	6,528 (48.7)	8.5 (7.5-9.5)	3.4 (2.7-4.1)	2.8 (2.2-3.5)
Boy	6,867 (51.3)	13.0 (11.7-14.2)	5.1 (4.4-5.8)	3.7 (3.0-4.4)
Diarrhea in past two weeks				
Yes	2,116 (15.8)	9.0 (7.4-10.6)	4.1 (2.9-5.4)	2.6 (1.7-3.5)
No	11,250 (84.0)	11.1 (10.2-12.0)	4.3 (3.7-4.8)	3.4 (2.9-3.9)
Missing	29 (0.2)	19.8 (2.8-36.7)	11.7 (0.0-26.4)	0.0 (0.0-0.0)
Fever in past two weeks				
Yes	2,732 (20.4)	11.8 (10.1-13.5)	5.8 (4.6-7.0)	3.8 (2.8-4.9)
No	10,633 (79.4)	10.5 (9.6-11.5)	3.9 (3.4-4.4)	3.1 (2.6-3.7)
Missing	30 (0.2)	5.5 (0.0-12.7)	2.0 (0.0-4.9)	0.0 (0.0-0.0)
Cough in past two weeks				
Yes	3,109 (23.2)	11.1 (9.5-12.7)	5.3 (4.1-6.5)	3.5 (2.6-4.4)
No	10,267 (76.6)	10.6 (9.7-11.6)	3.9 (3.4-4.5)	3.2 (2.7-3.8)
Missing	19 (0.1)	23.1 (0.0-49.2)	14.2 (0.0-39.1)	0.0 (0.0-0.0)
Mother's age				
15-19 years	1,589 (11.9)	12.8 (10.3-15.4)	4.1 (2.9-5.4)	4.3 (2.8-5.8)

20-34 years	9,743 (72.7)	10.4 (9.4-11.3)	4.2 (3.6-4.9)	2.9 (2.4-3.4)
35-49 years	2,063 (15.4)	11.0 (9.0-12.9)	4.5 (3.2-5.8)	4.2 (2.8-5.6)
Mother’s education level				
Primary or none	3,663 (27.3)	14.2 (12.8-15.6)	6.4 (5.4-7.4)	3.3 (2.4-4.1)
Secondary	6,775 (50.6)	9.8 (8.7-11.0)	3.9 (3.2-4.6)	3.5 (2.8-4.2)
Higher	2,688 (20.1)	8.6 (6.6-10.5)	2.1 (1.3-2.9)	2.6 (1.7-3.5)
Missing	269 (2.0)	14.0 (8.9-19.2)	9.5 (4.8-14.2)	5.3 (2.3-8.3)
Mother's marital or cohabitation status				
Yes	10,301 (76.9)	10.3 (9.4-11.2)	4.2 (3.6-4.7)	3.2 (2.7-3.7)
No	3,072 (22.9)	12.4 (10.6-14.2)	4.5 (3.4-5.7)	3.6 (2.6-4.6)
Missing	22 (0.2)	17.8 (0.0-42.9)	14.4 (0.0-39.5)	1.9 (0.0-5.7)
Mother ever consumed alcohol				
Yes	7,900 (59.0)	9.5 (8.5-10.5)	3.6 (3.0-4.2)	3.0 (2.4-3.5)
No	5,436 (40.6)	12.7 (11.5-14.0)	5.4 (4.5-6.2)	3.8 (3.0-4.6)
Missing	59 (0.4)	18.7 (6.9-30.5)	2.0 (0.0-5.0)	5.4 (0.0-11.8)
Experienced child death				
Yes	513 (3.8)	16.5 (11.9-21.1)	9.4 (5.4-13.5)	4.2 (1.7-6.8)
No	12,879 (96.1)	10.5 (9.7-11.4)	4.1 (3.6-4.6)	3.2 (2.8-3.7)
Missing	3 (0.0)	75.5 (24.3-100.0)	0.0 (0.0-0.0)	0.0 (0.0-0.0)
Household wealth quintile				
1, poorest	4,220 (31.5)	14.2 (12.6-15.7)	6.0 (5.0-7.1)	3.7 (2.7-4.7)
2	2,860 (21.4)	11.9 (10.0-13.8)	4.1 (3.1-5.1)	2.8 (1.8-3.7)
3	2,533 (18.9)	9.2 (7.6-10.9)	4.0 (2.9-5.0)	3.7 (2.6-4.7)
4	2,136 (15.9)	7.3 (5.5-9.1)	3.2 (2.2-4.2)	2.5 (1.6-3.3)
5, richest	1,646 (12.3)	9.1 (7.0-11.3)	2.9 (1.5-4.4)	3.8 (2.2-5.3)

Ever breastfed				
Yes	12,587 (94.0)	10.5 (9.7-11.4)	4.0 (3.5-4.5)	3.2 (2.7-3.6)
No	795 (5.9)	14.6 (11.1-18.1)	8.8 (5.7-11.9)	4.9 (2.8-7.0)
Missing	13 (0.1)	4.4 (0.0-13.1)	4.4 (0.0-13.1)	0.0 (0.0-0.0)
Safe disposal of child's stool				
Yes	3,352 (25.0)	10.6 (9.0-12.2)	3.9 (2.9-4.9)	3.3 (2.3-4.2)
No	10,021 (74.8)	10.8 (9.9-11.8)	4.4 (3.8-4.9)	3.3 (2.7-3.8)
Missing	22 (0.2)	7.2 (0.0-17.6)	4.6 (0.0-13.6)	10.7 (0.0-24.8)
Cesarean delivery				
Yes	4,938 (36.9)	10.6 (9.4-11.9)	3.3 (2.6-4.0)	2.0 (1.5-2.6)
No	7,715 (57.6)	10.2 (9.2-11.3)	4.4 (3.7-5.0)	4.0 (3.3-4.7)
Missing	742 (5.5)	17.7 (13.7-21.7)	9.7 (6.5-13.0)	4.2 (1.9-6.6)
Received antenatal care				
Yes	12,633 (94.3)	10.5 (9.7-11.4)	4.1 (3.6-4.6)	3.2 (2.8-3.7)
No	525 (3.9)	15.5 (10.8-20.2)	7.9 (4.0-11.9)	3.2 (1.6-4.8)
Missing	237 (1.8)	14.2 (6.5-21.9)	6.7 (0.6-12.8)	5.1 (0.0-10.7)
Breastfeeding guidance after delivery				
Yes	9,878 (73.7)	10.3 (9.3-11.2)	4.0 (3.4-4.5)	3.2 (2.6-3.7)
No	3,242 (24.2)	12.1 (10.6-13.6)	5.1 (4.2-6.1)	3.5 (2.5-4.5)
Missing	275 (2.1)	14.6 (7.6-21.5)	6.1 (0.6-11.6)	5.1 (0.1-10.2)

Note: CI, confidence interval.

Table S8. Odds Ratios (OR) and 95% Confidence Intervals (CI) for Stunting, Underweight, and Wasting by Selected Factors Among Children Aged 0-23 Months in Eight Countries or Regions of Latin America and the Caribbean

Factor	Stunting		Underweight		Wasting	
	OR (95% CI)	*P*-value	OR (95% CI)	*P*-value	OR (95% CI)	*P*-value
Children's age						
0-11 months	Ref		Ref		Ref	
12-23 months	1.51 (1.27-1.79)	<0.0001	1.08 (0.85-1.37)	0.5421	0.62 (0.46-0.85)	0.0026
Children's sex						
Boy	Ref		Ref		Ref	
Girl	0.61 (0.52-0.72)	<0.0001	0.64 (0.50-0.83)	0.0006	0.74 (0.55-1.01)	0.0547
Diarrhea in past 2 weeks						
No	Ref		Ref		Ref	
Yes	0.68 (0.55-0.85)	0.0005	0.76 (0.54-1.07)	0.1186	0.76 (0.51-1.14)	0.1819
Fever in past 2 weeks						
No	Ref		Ref		Ref	
Yes	1.11 (0.90-1.37)	0.3162	1.28 (0.96-1.72)	0.0958	1.22 (0.84-1.78)	0.2871
Cough in past 2 weeks						
No	Ref		Ref		Ref	
Yes	1.01 (0.82-1.23)	0.9557	1.22 (0.91-1.66)	0.1882	1.13 (0.80-1.60)	0.4863
Mother's age						
20-34 years	Ref		Ref		Ref	
15-19 years	1.19 (0.92-1.55)	0.1811	0.84 (0.59-1.19)	0.3352	1.42 (0.95-2.13)	0.0887
35-49 years	0.99 (0.79-1.25)	0.9567	1.00 (0.71-1.42)	0.9932	1.47 (0.97-2.22)	0.0695
Mother's education level						
Primary or none	Ref		Ref		Ref	
Secondary	0.86 (0.70-1.04)	0.1191	0.72 (0.53-0.98)	0.0357	0.83 (0.55-1.25)	0.3694

Higher	0.83 (0.61-1.15)	0.2632	0.48 (0.29-0.78)	0.0030	0.76 (0.46-1.27)	0.2975
Mother's marital or cohabitation status						
No	Ref		Ref		Ref	
Yes	0.85 (0.70-1.03)	0.0998	0.80 (0.59-1.09)	0.1645	0.73 (0.52-1.03)	0.0766
Mother ever consumed alcohol						
No	Ref		Ref		Ref	
Yes	0.86 (0.71-1.03)	0.0915	0.76 (0.57-1.01)	0.0595	0.72 (0.51-1.02)	0.0613
Experienced child death						
No	Ref		Ref		Ref	
Yes	1.53 (1.08-2.16)	0.0168	1.96 (1.19-3.24)	0.0084	1.30 (0.67-2.53)	0.4453
Household wealth quintile						
1, poorest	Ref		Ref		Ref	
2	0.84 (0.67-1.05)	0.1226	0.76 (0.55-1.04)	0.0815	0.86 (0.56-1.32)	0.4930
3	0.66 (0.52-0.84)	0.0007	0.79 (0.56-1.12)	0.1855	1.28 (0.85-1.92)	0.2329
4	0.52 (0.39-0.70)	<0.0001	0.64 (0.45-0.93)	0.0184	0.89 (0.57-1.40)	0.6179
5, richest	0.71 (0.52-0.98)	0.0358	0.72 (0.39-1.31)	0.2813	1.50 (0.91-2.47)	0.1156
Ever breastfed						
No	Ref		Ref		Ref	
Yes	0.72 (0.53-0.96)	0.0267	0.50 (0.34-0.74)	0.0005	0.66 (0.42-1.06)	0.0835
Safe disposal of child's stool						
No	Ref		Ref		Ref	
Yes	1.12 (0.92-1.35)	0.2478	1.04 (0.77-1.40)	0.8193	1.20 (0.77-1.87)	0.4134
Cesarean delivery						
No	Ref		Ref		Ref	
Yes	1.14 (0.93-1.40)	0.2225	0.98 (0.73-1.31)	0.8760	0.54 (0.38-0.77)	0.0005
Received antenatal care						

No	Ref		Ref		Ref	
Yes	0.67 (0.47-0.97)	0.0317	0.71 (0.42-1.21)	0.2047	1.52 (0.85-2.71)	0.1594
Breastfeeding guidance after delivery						
No	Ref		Ref		Ref	
Yes	0.99 (0.83-1.19)	0.9504	0.98 (0.77-1.26)	0.8864	0.84 (0.59-1.20)	0.3427

Note: OR, odds ratio; CI, confidence interval.

Table S9. Prevalence of Stunting, Underweight, and Wasting by Selected Factors Among Children Aged 0–59 Months in Seven Countries or Regions of Latin America and the Caribbean Excluding Cuba

Factor	Children observed, No. (%)	Prevalence, %(95%CI)		
		Stunting	Underweight	Wasting
Total	29,768 (100.0)	11.4 (10.8-12.0)	4.8 (4.4-5.2)	2.8 (2.5-3.1)
Children's age				
0-11 months	5,523 (18.6)	9.4 (8.3-10.6)	4.5 (3.7-5.3)	4.1 (3.3-5.0)
12-23 months	6,009 (20.2)	13.3 (12.0-14.6)	4.9 (4.1-5.7)	2.7 (2.1-3.2)
24-35 months	5,901 (19.8)	12.7 (11.5-13.8)	5.3 (4.5-6.0)	2.5 (1.9-3.2)
36-47 months	6,269 (21.1)	11.9 (10.7-13.2)	4.9 (4.1-5.6)	2.1 (1.6-2.6)
48-59 months	6,066 (20.4)	9.5 (8.5-10.6)	4.5 (3.7-5.3)	2.5 (1.9-3.0)
Children's sex				
Girl	14,610 (49.1)	10.6 (9.8-11.3)	4.2 (3.8-4.7)	2.4 (2.0-2.7)
Boy	15,158 (50.9)	12.2 (11.3-13.0)	5.4 (4.9-5.9)	3.2 (2.8-3.6)
Diarrhea in past two weeks				
Yes	3,886 (13.1)	10.0 (8.8-11.3)	4.7 (3.7-5.8)	2.8 (2.0-3.5)
No	25,810 (86.7)	11.6 (10.9-12.2)	4.8 (4.4-5.2)	2.8 (2.5-3.1)
Missing	72 (0.2)	13.7 (4.9-22.6)	6.0 (0.0-12.2)	0.8 (0.0-2.5)
Fever in past two weeks				
Yes	6,267 (21.1)	11.1 (10.1-12.2)	6.1 (5.2-6.9)	3.6 (2.9-4.2)
No	23,442 (78.7)	11.4 (10.7-12.1)	4.5 (4.1-4.9)	2.6 (2.3-2.9)
Missing	59 (0.2)	16.0 (0.1-31.9)	9.4 (0.0-24.9)	0.0 (0.0-0.0)
Cough in past two weeks				
Yes	7,752 (26.0)	10.6 (9.7-11.6)	5.3 (4.5-6.1)	2.9 (2.4-3.4)
No	21,968 (73.8)	11.6 (10.9-12.4)	4.6 (4.2-5.0)	2.7 (2.4-3.1)

Missing	48 (0.2)	16.7 (1.1-32.4)	5.0 (0.0-14.5)	1.6 (0.0-4.1)
Mother’s age				
15-19 years	2,078 (7.0)	14.1 (11.8-16.5)	4.8 (3.6-6.0)	3.5 (2.3-4.6)
20-34 years	21,206 (71.2)	11.3 (10.6-12.0)	4.7 (4.3-5.2)	2.6 (2.3-2.9)
35-49 years	6,484 (21.8)	10.9 (9.7-12.1)	5.1 (4.3-5.9)	3.2 (2.6-3.9)
Mother’s education level				
Primary or none	9,555 (32.1)	17.2 (16.1-18.3)	7.0 (6.3-7.7)	2.5 (2.0-2.9)
Secondary	13,489 (45.3)	9.6 (8.8-10.5)	4.4 (3.8-4.9)	3.1 (2.7-3.6)
Higher	5,928 (19.9)	6.1 (5.1-7.2)	2.2 (1.7-2.8)	2.4 (1.8-3.0)
Missing	796 (2.7)	16.9 (13.3-20.5)	7.9 (5.1-10.6)	2.3 (1.2-3.3)
Mother's marital or cohabitation status				
Yes	22,682 (76.2)	11.3 (10.6-12.0)	4.8 (4.4-5.2)	2.8 (2.4-3.1)
No	7,040 (23.6)	11.6 (10.4-12.9)	4.7 (4.0-5.5)	2.9 (2.3-3.4)
Missing	46 (0.2)	9.1 (0.0-23.0)	7.3 (0.0-21.0)	2.9 (0.0-7.3)
Mother ever consumed alcohol				
Yes	16,545 (55.6)	8.9 (8.1-9.6)	4.0 (3.6-4.5)	2.8 (2.4-3.2)
No	10,335 (34.7)	16.1 (15.0-17.1)	6.6 (6.0-7.3)	2.9 (2.5-3.4)
Missing	2,888 (9.7)	10.8 (8.6-13.0)	3.3 (1.9-4.7)	2.2 (1.2-3.1)
Experienced child death				
Yes	1,486 (5.0)	17.0 (14.1-19.8)	7.7 (5.8-9.7)	3.0 (1.8-4.2)
No	28,270 (95.0)	11.1 (10.5-11.7)	4.7 (4.3-5.0)	2.8 (2.5-3.1)
Missing	12 (0.0)	22.1 (0.0-48.6)	0.0 (0.0-0.0)	0.0 (0.0-0.0)
Household wealth quintile				
1, poorest	9,542 (32.1)	16.8 (15.4-18.1)	6.8 (6.0-7.5)	2.7 (2.2-3.2)
2	6,594 (22.2)	11.8 (10.6-12.9)	5.0 (4.2-5.7)	2.6 (2.0-3.2)

3	5,593 (18.8)	9.4 (8.3-10.5)	4.3 (3.5-5.1)	3.3 (2.6-4.1)
4	4,577 (15.4)	7.6 (6.4-8.7)	3.6 (2.9-4.4)	2.6 (2.0-3.2)
5, richest	3,462 (11.6)	7.5 (6.0-8.9)	2.8 (1.9-3.6)	2.7 (1.9-3.5)
Child health insurance coverage				
Yes	12,835 (43.1)	7.6 (6.9-8.4)	3.5 (3.0-3.9)	2.6 (2.2-3.0)
No	16,910 (56.8)	14.4 (13.6-15.3)	5.9 (5.4-6.5)	2.9 (2.5-3.3)
Missing	23 (0.1)	10.2 (0.0-22.1)	5.2 (0.0-15.3)	0.0 (0.0-0.0)
Mother's health insurance coverage				
Yes	13,199 (44.3)	7.4 (6.7-8.1)	3.2 (2.8-3.7)	2.5 (2.1-2.9)
No	16,546 (55.6)	14.8 (13.9-15.6)	6.1 (5.6-6.7)	3.0 (2.6-3.4)
Missing	23 (0.1)	2.7 (0.0-6.9)	0.0 (0.0-0.0)	0.0 (0.0-0.0)

Note: CI, confidence interval.

Table S10. Odds Ratios (OR) and 95% Confidence Intervals (CI) for Stunting, Underweight, and Wasting by Selected Factors Among Children Aged 0-59 Months in Seven Countries or Regions of Latin America and the Caribbean Excluding Cuba

Factor	Stunting		Underweight		Wasting	
	OR (95% CI)	*P*-value	OR (95% CI)	*P*-value	OR (95% CI)	*P*-value
Children's age						
0-11 months	Ref		Ref		Ref	
12-23 months	1.55 (1.30-1.85)	<0.0001	1.09 (0.86-1.39)	0.4812	0.63 (0.47-0.85)	0.0026
24-35 months	1.43 (1.21-1.69)	<0.0001	1.17 (0.92-1.47)	0.1979	0.57 (0.41-0.79)	0.0008
36-47 months	1.31 (1.10-1.57)	0.0027	1.07 (0.84-1.35)	0.5867	0.51 (0.38-0.69)	<0.0001
48-59 months	1.11 (0.92-1.35)	0.2846	1.08 (0.83-1.41)	0.5520	0.59 (0.44-0.79)	0.0005
Children's sex						
Boy	Ref		Ref		Ref	
Girl	0.85 (0.76-0.94)	0.0025	0.81 (0.70-0.94)	0.0051	0.76 (0.63-0.93)	0.0059
Diarrhea in past 2 weeks						
No	Ref		Ref		Ref	
Yes	0.83 (0.71-0.96)	0.0131	0.93 (0.74-1.19)	0.5801	0.92 (0.68-1.24)	0.5728
Fever in past 2 weeks						
No	Ref		Ref		Ref	
Yes	1.08 (0.94-1.24)	0.2571	1.36 (1.13-1.63)	0.0010	1.44 (1.12-1.87)	0.0051
Cough in past 2 weeks						
No	Ref		Ref		Ref	
Yes	0.92 (0.81-1.04)	0.1865	1.05 (0.87-1.28)	0.6051	0.98 (0.76-1.26)	0.8583
Mother's age						
20-34 years	Ref		Ref		Ref	
15-19 years	1.05 (0.85-1.30)	0.6426	0.84 (0.62-1.14)	0.2623	1.34 (0.92-1.96)	0.1244
35-49 years	0.94 (0.81-1.08)	0.3601	1.04 (0.86-1.25)	0.7108	1.29 (1.01-1.64)	0.0402

Mother's education level						
Primary or none	Ref		Ref		Ref	
Secondary	0.81 (0.70-0.93)	0.0023	0.74 (0.61-0.90)	0.0027	0.93 (0.71-1.22)	0.6009
Higher	0.65 (0.53-0.80)	<0.0001	0.51 (0.36-0.71)	<0.0001	0.84 (0.58-1.22)	0.3579
Mother's marital or cohabitation status						
No	Ref		Ref		Ref	
Yes	1.00 (0.88-1.14)	0.9853	0.91 (0.75-1.09)	0.2911	0.77 (0.62-0.97)	0.0254
Mother ever consumed alcohol						
No	Ref		Ref		Ref	
Yes	0.86 (0.76-0.99)	0.0293	0.82 (0.68-0.98)	0.0335	0.78 (0.62-0.98)	0.0360
Experienced child death						
No	Ref		Ref		Ref	
Yes	1.45 (1.17-1.80)	0.0007	1.42 (1.05-1.91)	0.0234	1.12 (0.73-1.71)	0.6173
Household wealth quintile						
1, poorest	Ref		Ref		Ref	
2	0.70 (0.60-0.81)	<0.0001	0.83 (0.69-1.00)	0.0512	1.13 (0.85-1.50)	0.4068
3	0.56 (0.48-0.66)	<0.0001	0.72 (0.58-0.90)	0.0045	1.57 (1.18-2.10)	0.0022
4	0.52 (0.42-0.63)	<0.0001	0.67 (0.53-0.85)	0.0009	1.22 (0.91-1.65)	0.1891
5, richest	0.62 (0.48-0.80)	0.0002	0.76 (0.53-1.08)	0.1285	1.54 (1.08-2.19)	0.0165
Child health insurance coverage						
No	Ref		Ref		Ref	
Yes	0.85 (0.69-1.05)	0.1372	0.96 (0.73-1.26)	0.7721	0.99 (0.73-1.35)	0.9376
Mother's health insurance coverage						
No	Ref		Ref		Ref	
Yes	0.96 (0.80-1.15)	0.6512	0.86 (0.66-1.12)	0.2701	0.74 (0.56-0.97)	0.0298

Note: OR, odds ratio; CI, confidence interval.

Table S11. Odds Ratios (OR) and 95% Confidence Intervals (CI) for Stunting, Underweight, and Wasting by Selected Factors Among Children Aged 0-59 Months in Seven Countries or Regions of Latin America and the Caribbean Excluding Turks and Caicos Islands

Factor	Stunting		Underweight		Wasting	
	OR (95% CI)	*P*-value	OR (95% CI)	*P*-value	OR (95% CI)	*P*-value
Children's age						
0-11 months	Ref		Ref		Ref	
12-23 months	1.57 (1.32-1.87)	<0.0001	1.09 (0.86-1.39)	0.4685	0.64 (0.47-0.86)	0.0033
24-35 months	1.45 (1.23-1.71)	<0.0001	1.17 (0.92-1.48)	0.1952	0.58 (0.41-0.80)	0.0012
36-47 months	1.33 (1.12-1.58)	0.0015	1.07 (0.84-1.35)	0.5947	0.51 (0.38-0.70)	<0.0001
48-59 months	1.13 (0.93-1.36)	0.2168	1.08 (0.83-1.41)	0.5534	0.59 (0.43-0.79)	0.0005
Children's sex						
Boy	Ref		Ref		Ref	
Girl	0.84 (0.75-0.94)	0.0017	0.81 (0.70-0.94)	0.0051	0.77 (0.63-0.93)	0.0065
Diarrhea in past 2 weeks						
No	Ref		Ref		Ref	
Yes	0.83 (0.71-0.96)	0.0129	0.93 (0.74-1.19)	0.5741	0.92 (0.68-1.23)	0.5604
Fever in past 2 weeks						
No	Ref		Ref		Ref	
Yes	1.08 (0.95-1.24)	0.2397	1.36 (1.13-1.63)	0.0010	1.45 (1.12-1.88)	0.0046
Cough in past 2 weeks						
No	Ref		Ref		Ref	
Yes	0.92 (0.81-1.05)	0.2075	1.05 (0.86-1.28)	0.6083	0.98 (0.76-1.26)	0.8570
Mother's age						
20-34 years	Ref		Ref		Ref	
15-19 years	1.07 (0.87-1.32)	0.5296	0.85 (0.63-1.14)	0.2718	1.36 (0.94-1.97)	0.1076
35-49 years	0.93 (0.81-1.07)	0.3365	1.03 (0.85-1.24)	0.7811	1.27 (1.00-1.62)	0.0536

Mother's education level						
Primary or none	Ref		Ref		Ref	
Secondary	0.79 (0.69-0.90)	0.0007	0.72 (0.59-0.88)	0.0014	0.89 (0.68-1.18)	0.4294
Higher	0.62 (0.51-0.76)	<0.0001	0.48 (0.35-0.67)	<0.0001	0.77 (0.54-1.12)	0.1738
Mother's marital or cohabitation status						
No	Ref		Ref		Ref	
Yes	1.00 (0.87-1.14)	0.9809	0.90 (0.75-1.08)	0.2562	0.76 (0.60-0.95)	0.0170
Mother ever consumed alcohol						
No	Ref		Ref		Ref	
Yes	0.86 (0.76-0.98)	0.0205	0.83 (0.69-0.99)	0.0430	0.78 (0.62-0.98)	0.0356
Experienced child death						
No	Ref		Ref		Ref	
Yes	1.46 (1.18-1.81)	0.0006	1.42 (1.05-1.92)	0.0221	1.11 (0.73-1.71)	0.6218
Household wealth quintile						
1, poorest	Ref		Ref		Ref	
2	0.69 (0.60-0.80)	<0.0001	0.82 (0.68-0.99)	0.0380	1.09 (0.82-1.46)	0.5385
3	0.55 (0.47-0.65)	<0.0001	0.71 (0.57-0.88)	0.0021	1.51 (1.13-2.02)	0.0056
4	0.50 (0.41-0.61)	<0.0001	0.65 (0.51-0.82)	0.0003	1.16 (0.86-1.57)	0.3283
5, richest	0.60 (0.47-0.76)	<0.0001	0.73 (0.51-1.04)	0.0777	1.44 (1.01-2.03)	0.0410

Note: OR, odds ratio; CI, confidence interval. Turks and Caicos Islands was excluded from these models due to its small sample size to ensure that the overall pooled estimates were not unduly biased by sparse data strata. Country fixed effects were included in all models.

Table S12. Ratio of Odds Ratios (ROR) and 95% Confidence Intervals (CI) for the Interactions of Diarrhea by Age on Stunting

Model and Interaction Term	ROR(95% CI)	*P*-value
Diarrhea (Yes) × 0–11 months	Ref	
Diarrhea (Yes) × 12–23 months	1.21 (0.76-1.93)	0.4295
Diarrhea (Yes) × 24–35 months	1.93 (1.20-3.10)	0.0070
Diarrhea (Yes) × 36–47 months	1.42 (0.86-2.34)	0.1709
Diarrhea (Yes) × 48–59 months	1.26 (0.70-2.29)	0.4442

Note: ROR, ratio of odds ratios; CI, confidence interval. This table reports the exponentiated coefficients of the product terms added to the fully adjusted main pooled model, testing for multiplicative interaction between diarrhea and age group on stunting. An ROR of 1.93 indicates that the odds ratio of stunting associated with recent diarrhea in the 24–35 months age group is 1.93 times higher than the corresponding odds ratio in the 0–11 months reference group. This significant interaction (*P* = 0.0070) suggests that the association between diarrhea and stunting varies by age, with a stronger positive association observed during the 24–35 months age period—a critical weaning window—which contrasts with the unexpected inverse association seen in the unstratified main model.

Figure S1. Diagnostic trace plots for convergence of the multiple imputation by chained equations (MICE) procedure

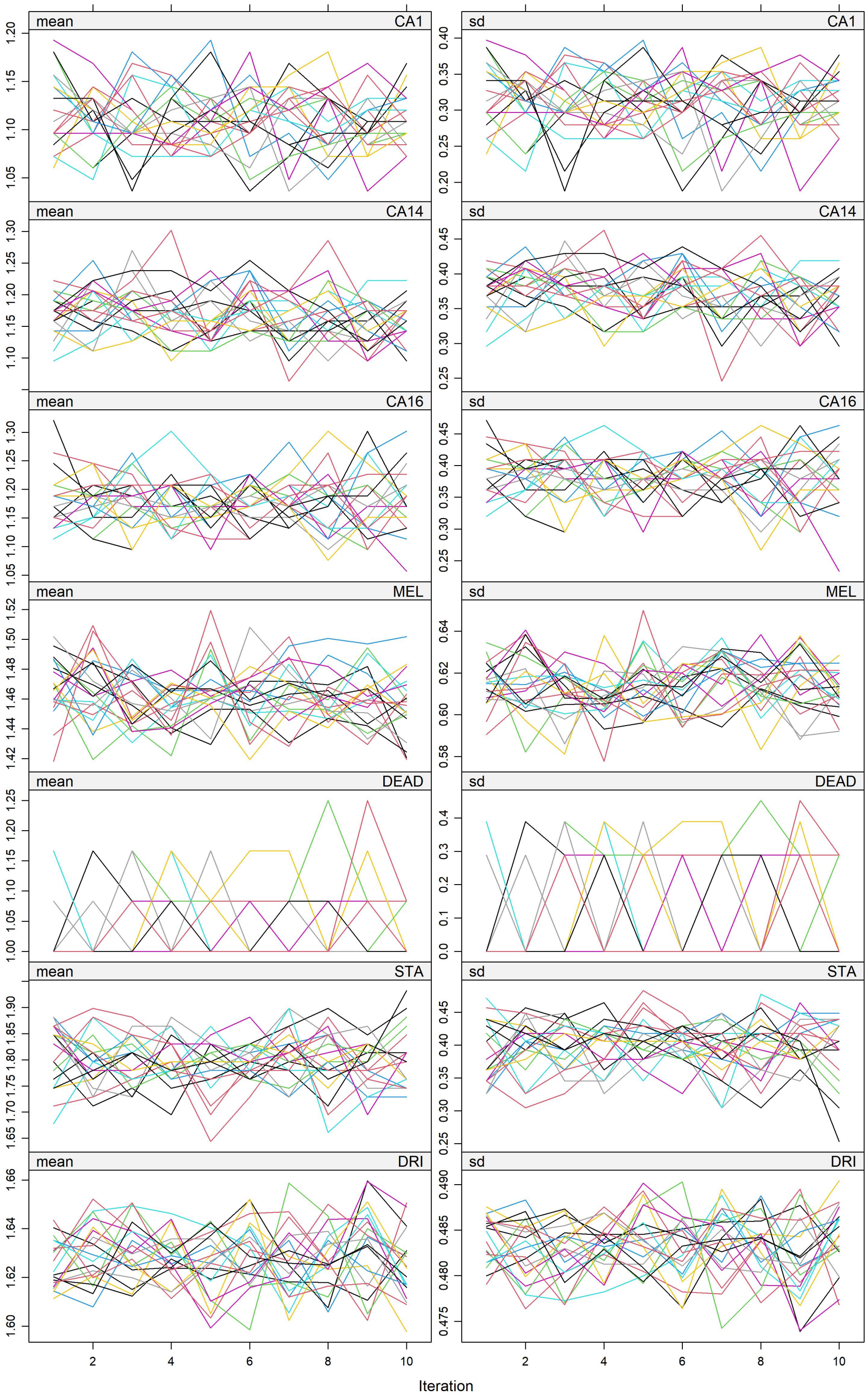

Note: The plots display the iteration-specific mean (left panels) and standard deviation (right panels) for the imputed variables across the 20 multiple imputed datasets over 10 iterations. Variable abbreviations correspond to the following variables: CA1 (Diarrhea in past 2 weeks); CA14 (Fever in past 2

weeks); CA16 (Cough in past 2 weeks); MEL (Mother's education level); DEAD (Whether the mother has deceased children); STA (Mother’s marital or cohabitation status); DRI (Mother ever consumed alcohol). For most variables, the stable mixing of the 20 imputation chains (represented by different colors) and the absence of systematic trends over the iterations indicate satisfactory convergence of the imputation algorithm. Of note, the trace plot for “DEAD” exhibits a discontinuous, jagged pattern with intermittent zero standard deviations; this is an expected mathematical artifact driven by its extremely low missingness rate (< 0.1%). Because only a negligible number of observations are being imputed in a binary space, any minor shift in assignment during an iteration causes dramatic relative jumps in the imputed subset's mean and variance. This discrete imputation behavior for near-complete variables does not affect the overall stability or validity of the pooled main models.

Figure S2. Odds Ratios (OR) and 95% Confidence Intervals (CI) for Stunting, Underweight, and Wasting by Maternal Education Level and Household Wealth Quintile Among Children Aged 0-59 Months in Seven Countries or Regions of Latin America and the Caribbean Excluding Turks and Caicos Islands

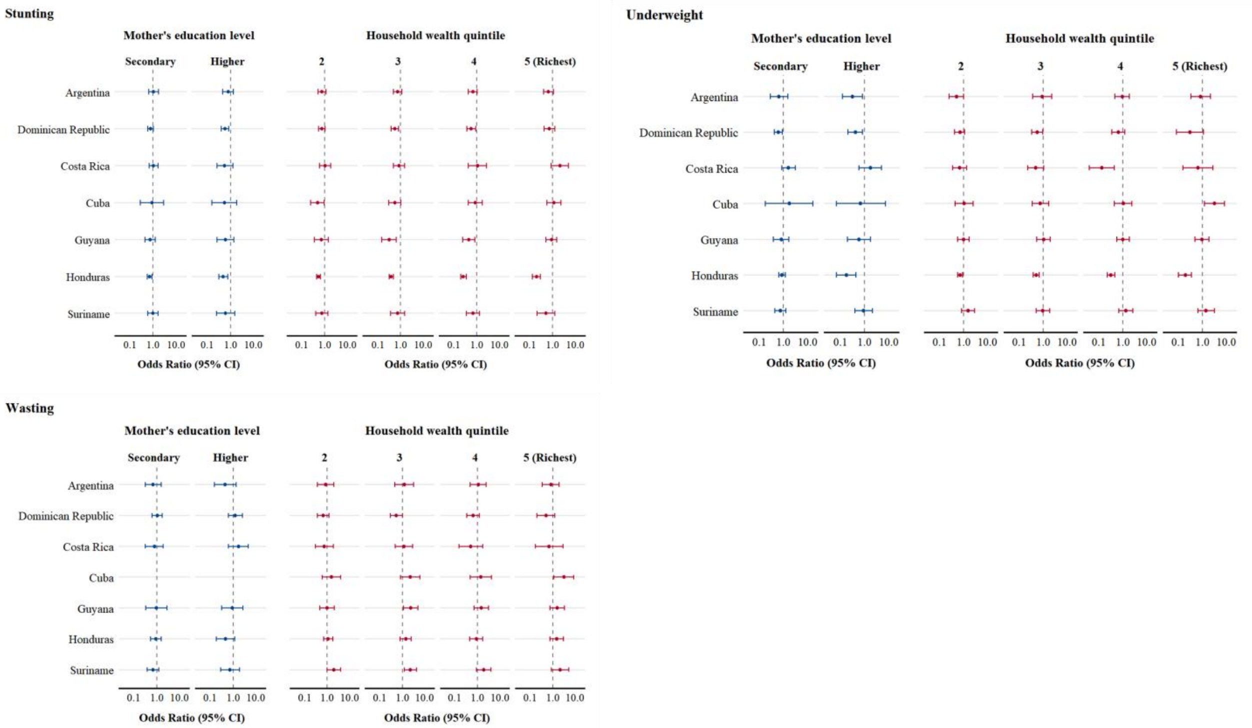

Note: Blue circles represent odds ratios (OR) for maternal education levels, with "Primary or none" as the reference group. Red circles represent OR for household wealth quintiles, with the "1 (poorest)" quintile as the reference group. Horizontal error bars indicate 95% CI, and the vertical dashed lines represent an OR of 1.0. To ensure stable mathematical convergence within individual country strata, all models were minimally adjusted for children's age and sex. Turks and Caicos Islands was excluded from this visualization due to insufficient overall sample size and case counts. In the wasting model for Cuba, the OR for maternal education

were not estimable (left blank in the plot) due to the statistical phenomenon of complete separation; this was driven by a nearly empty reference group (caused by the country's universally high education levels) coupled with an extremely low prevalence of wasting.
